# Refractive development III: Variations in emmetropia and ametropia

**DOI:** 10.1111/opo.13516

**Published:** 2025-05-19

**Authors:** Jos J. Rozema, Mohammad Hassan Emamian, Hassan Hashemi, Akbar Fotouhi

**Affiliations:** ^1^ Visual Optics Lab Antwerp (VOLANTIS), Faculty of Medicine and Health Sciences University of Antwerp Wilrijk Belgium; ^2^ Department of Ophthalmology Antwerp University Hospital Edegem Belgium; ^3^ Institute for Medical Informatics, Statistics and Epidemiology (IMISE), Leipzig University Leipzig Germany; ^4^ Ophthalmic Epidemiology Research Centre Shahroud University of Medical Sciences Shahroud Iran; ^5^ Noor Research Centre for Ophthalmic Epidemiology Noor Eye Hospital Tehran Iran; ^6^ Department of Epidemiology and Biostatistics School of Public Health, Tehran University of Medical Sciences Tehran Iran

**Keywords:** ametropia, bigaussian analysis, emmetropia, ocular biometry

## Abstract

**Purpose:**

The purpose of this study was to investigate biometric variations in emmetropia and ametropia, focusing on the differences and correlations found in adult eyes. Bigaussian analyses were performed to characterise the biometric properties of the Regulated (or Emmetropised) and Dysregulated subpopulations identified in earlier works.

**Methods:**

This work analyses the biometric and refractive error data of 2000 adult participants previously recruited during the first phase of the Shahroud Eye Cohort Study (Iran). Measurements included cycloplegic subjective refractive error, corneal radii of curvature and intraocular distances. The study employed multivariate bigaussian fits and statistical analyses to explore the relationships between ocular biometry parameters.

**Results:**

Significant correlations were found between ocular dimensions, suggesting the existence of ocular shape factors. Meanwhile, there was a large overlap in biometric values across refractive groups, especially within ±3 D. The 979 emmetropic eyes, defined as having a refractive error exceeding ±0.5 D, had axial lengths ranging between 20.86 and 25.62 mm, with matching corneal and lens powers that together ensured emmetropia. The distribution of both the refractive errors and the ocular biometry parameters was leptokurtic and skewed, and could be described accurately by a bigaussian function, representing the Regulated and Dysregulated subpopulations. Regulated eyes had well‐matched biometric parameters, while in Dysregulated eyes one or more deviations were seen.

**Conclusion:**

The terms refractive and axial ametropia are inadequate given the many interactions between ocular dimensions during eye growth, while emmetropisation is largely determined by the ability of the crystalline lens to lose power as the eye grows. ‘Regulated’ and ‘Dysregulated’ eyes are suitable alternatives, distinguished based on their relative biometry within a population. While the biometry of each eye is unique, large overlaps exist in the values of different refractive groups. These observations underscore the importance of using comprehensive rather than partial biometric data when studying or managing refractive errors.

**Key points**

- The many significant correlations between the ocular dimensions point to the existence of ocular shape factors.
- These shape factors may be linked to the Regulated and Dysregulated subgroups defined through bigaussian analysis.
- The large overlap in biometric ranges between refractive groups means that partial information (e.g., just refractive error and axial length) is insufficient to characterise the refractive condition of an eye.

## INTRODUCTION

To understand the aetiology of refractive error and refractive development, one must first understand where emmetropia comes from, as it forms an essential reference by which to judge anomalies. Although this observation by Donders^1^ in 1864 is seemingly obvious, emmetropia is not trivial, but rather the result of a complex choreography of eye growth.^2^, ^3^

It was known before the 19th century that in emmetropic eyes, the focal point of a parallel light beam entering a distance‐focused eye is located on the fovea, while in hypermetropia and myopia it falls behind the retina and in the vitreous, respectively. Although the optics of emmetropia and ametropia have been described in detail by great names such as Young^4^ and Listing,^5^ it took nearly a century until theories on refractive development emerged. These began in 1909 with Straub,^6^ who noticed that the refractive distribution in newborns was broad and centred on low hypermetropia, whereas in adults it was narrower with a large portion of emmetropic eyes. From this, he deduced that there must be a growth process to focus the eye that he termed emmetropisation. Around the same time, Steiger^7^ reported that corneal power is normally distributed, from which he hypothesised that all other ocular dimensions may vary freely according to normal distributions and that refractive errors result from coincidental mismatches between them. Tron^8^ and Stenström^9^ later confirmed that other ocular dimensions were indeed normally distributed, including axial length (AL), provided highly myopic eyes were excluded. However, in order for this to be true, the distribution of refractive errors should be normal as well, which was at odds with observations of a leptokurtic and skewed distribution.^1^, ^9^, ^10^, ^11^, ^12^, ^13^, ^14^ Steiger was aware of this leptokurtosis and briefly remarked that this could be related to what he called a ‘directed variability’ of the corneal power and AL.^7^ While contradictory to his initial idea of a free association among ocular elements, directed variability is probably best understood as correlations between elements, Steiger presumed that longer eyes should have flatter corneas. Such biometric correlations were indeed confirmed a few decades later by Berg,^13^ Wibaut^15^ and especially Stenström,^9^ who during World War II explored the variations and correlations between ocular biometry measurements. Stenström used data from 1000 adults aged between 20 and 35 years, which was a tremendous undertaking, given the equipment available at the time, to provide researchers with the first complete overview of the relationship between the ocular components.^16^

Building on the work of these early pioneers, Sorsby et al. wrote a series of papers^17^, ^18^, ^19^, ^20^, ^21^ that comprehensively redefined the field of refractive development. These studies linked the correlations between the ocular components, or lack thereof,^17^, ^20^ to the development of refractive error and confirmed their importance in normal emmetropisation using a stochastic model.^18^ Some of their most noteworthy results included the observations that emmetropia can occur for a large range of ALs, that is, between 21 and 26 mm,^17^ while ocular components can have similar average values for refractive errors between –4 D and +6 D.^17^, ^20^ Furthermore, they found no correlation between corneal power and AL for eyes with refractive errors between –3 D and +3 D. Additionally, hypermetropic eyes had lower corneal powers than emmetropic eyes, while myopic eyes had higher values.^17^ Subsequent longitudinal analyses^19^, ^21^ showed that during eye growth, the large increase in AL was compensated by a decrease in corneal and, especially, lenticular power. Based on these observations, Sorsby et al. suggested that refractive errors could be divided into correlation ametropia, where all elements remained within the emmetropic range but were poorly matched, and component ametropia, where one of the elements (typically the AL) lay outside the emmetropic range.^17^ Although Sorsby et al. never clearly defined their concept of correlation, Carroll^22^, ^23^ demonstrated that a purely statistical interpretation of this term would not make sense. Instead, Carroll suggested interpreting this form of correlation as the ability of the refractive components to compensate adequately for a wide range of ALs.

It should be noted that up to the early 1960s, researchers often had to build their own measuring equipment. This changed with the introduction of clinical ultrasound devices, allowing others to confirm these early results, as performed by Gernet,^24^ Franceschetti and Luyckx,^25^ Larsen^26^, ^27^, ^28^, ^29^ and Francois and Goes.^30^ In more recent years, thousands of studies have been conducted, providing biometric data for populations of all ages and many ethnicities, including healthy and pathological eyes.

Over time, the interest in adult emmetropia seems to have waned and, much as Donders^1^ suggested, emmetropic eyes are nowadays mostly considered as the reference by which to judge pathology (sometimes unjustly so^31^). Meanwhile, the onset of the worldwide boom in myopia^32^ has understandably caused research efforts to be redirected towards investigating myopisation, yielding important new insights into how retinal feedback modulates the growth rates of the ocular elements during refractive development (see^2^, ^3^, ^33^, ^34^ for an overview).

The lack of recent studies on adult emmetropia may have given some clinicians the impression that the condition is well understood, while it remains unclear how eyes with very similar biometric values can end up having very different refractive errors on both sides of emmetropia. One possible direction in which the answer to this question could be found may be the observation of Flitcroft,^35^ who pointed out that the leptokurtic refractive distribution can be described as the sum of two overlapping Gaussian functions that together form a ‘bigaussian’ distribution. These, Flitcroft proposed, represent two populations defined by their refractive development that he called the Emmetropised (here named Regulated), that is, those where the emmetropisation process went as intended, and the Dysregulated, those where something altered this process along the way (e.g., hypermetropic stragglers or myopes). This is reminiscent of the idea proposed by Berg^13^ that eyes with emmetropia, hypermetropia and myopia are three distinct biological groups. Note that he considered the boundaries of the emmetropic group between +4 D and –3 D. Around the same time as Flitcroft, Rozema et al.^36^ presented a similar analysis that extended the bigaussian description to ocular biometry, confirming that the eyes in the two subpopulations have clearly different statistical properties. While the idea of bigaussian refractive error distributions is slowly being accepted,^37^ and was recently confirmed,^38^ not much other work has been done on this topic since then.

This work reassesses the biometric variations found in adult emmetropic and ametropic eyes to delineate their distinctions. As many original works in the field are not readily available, and often not available in English, this analysis starts from first principles, reproducing Sorsby et al.'s classic results on the overlapping distributions and correlations of the ocular elements, before introducing new observations on bigaussian distributions and the biometric nature of Regulated and Dysregulated eyes.

## MATERIALS AND METHODS

### Data

This analysis used the adult ocular biometry and refractive error data collected in the first phase of the Shahroud Eye Cohort Study (ShECS), details of which have been described elsewhere.^39^ In brief, ShECS aimed to determine the prevalence and incidence of visual impairment and major eye conditions in the Iranian city of Shahroud. Using random cluster sampling, inhabitants were invited for examination, of whom 82.2% consented. The examinations relevant to the current analysis included subjective refractive error under cycloplegia (one drop of 1% cyclopentolate and one drop of 2.5% phenylephrine instilled twice, 5 min apart), corneal radii of curvature using a Pentacam (Oculus, pentacam.com) and intraocular distances obtained using an Allegro Biograph (ALCON WaveLight, myalcon.com). ShECS was approved by the Shahroud University of Medical Sciences Ethics Committee and all participants signed an informed consent form prior to participating.

The data of the 5190 available participants was screened to exclude eyes with any current or previous ocular pathology (including cataract) or history of surgery, refractive error outside ±15 D or missing data for any parameter being considered. Moreover, only the right eyes were considered to avoid the influence of the strong correlation between the two eyes of an individual. After the screening, a total of 2000 right eyes of 2000 volunteers (38.7% male, 61.3% female), mean age 49.2 ± 5.7 years (range [39 to 64] years) were retained for further analysis. These eyes were considered emmetropic if their spherical equivalent (SE) refractive error was −0.5 D ≤ SE ≤ +0.5 D and perfectly emmetropic if SE = 0 D; all others were considered ametropic (myopia for SE < −0.5 D and hypermetropia for SE > +0.5 D).

### Biometry and calculations

The original dataset provided values for central corneal thickness (CCT), aqueous depth (ACD), lens thickness (LT) and AL as measured with the Biograph; the anterior and posterior corneal radii of curvature, r_ca_ and r_cp_, determined with the Pentacam and the subjective cycloplegic SE refractive error. These measured parameters formed the basis for the calculation of several others, such as the total corneal power P_c_, which was determined using the thick lens equation and the crystalline lens power P_l_, estimated using Bennett's equation,^40^, ^41^ which assumes that the lens has the same proportions as that of the Gullstrand–Emsley^42^ eye model. This approach was required as no phakometry measurements were available for this cohort. The equations needed to estimate these parameters are listed in Table 1 (their derivation can be found in Fincham and Freeman^43^).

**TABLE 1:** Parameters used in the models.

Symbol	Unit	Calculation/Value	Description
CCT	mm	Measured by Biograph	Central corneal thickness
AD	mm	Measured by Biograph	Aqueous depth (post. cornea to lens)
ACD_tot_	mm	CCT + AD	Anterior chamber depth (incl. cornea)
LT	mm	Measured by Biograph	Lens thickness
ASL	mm	ACD_tot_ + LT	Anterior segment length
VCD	mm	AL – ASL	Vitreous chamber depth
AL	mm	Measured by Biograph	Axial length
$n_{\mathrm{air}}$	–	1.000	Refractive index of air
$n_{c}$	–	1.376	Refractive index of the cornea
n	–	4/3	Refractive index of the ocular humours
$r_{\mathrm{ca}}$	mm	Measured by Pentacam	Anterior corneal radius of curvature
$r_{\mathrm{cp}}$	mm	Measured by Pentacam	Posterior corneal radius of curvature
$P_{\mathrm{ca}}$	D	$1000∙\left(n_{c}-n_{\mathrm{air}}\right)/r_{\mathrm{ca}}$	Anterior corneal curvature
$P_{\mathrm{cp}}$	D	$1000∙\left(n-n_{c}\right)/r_{\mathrm{cp}}$	Posterior corneal curvature
$P_{c}$	D	P_ca_ + P_cp_ – 0.001∙P_ca_∙P_cp_∙CCT/n_c_	Total corneal keratometry
P_cr_	–	P_c_/P_eye_	Corneal contribution to whole eye power
${\mathrm{pp}}_{c2}$	mm	–CCT ∙ n_a_/n_c_ ∙ P_ca_/P_c_	From ant. cornea to second corneal pp
$P_{\mathrm{lb}}$	D	Bennett's equation	Bennett's estimate of lens power^40^, ^41^
${\mathrm{pp}}_{l1}$	mm	0.571·LT	From ant. lens to first lenticular pp^41^
${\mathrm{pp}}_{l2}$	mm	−0.378·LT	From post. lens to second lenticular pp^41^
P_eye_	D	P_c_ + P_l_ – 0.001∙P_c_∙P_l_∙(pp_c2_+ ACD_tot_ + pp_l1_)/n	Whole eye power
${\mathrm{pp}}_{\mathrm{eye}2}$	mm	ASL – (pp_c2_ + ACD_tot_ + pp_l1_) ∙ n_v_/n_a_ ∙ P_c_/P_eye_	From ant. cornea to second ocular pp
P_ax_	D	1000∙n/(L – pp_eye2_)	Axial power in dioptre/dioptric distance
SE	D	P_ax_ – P_eye_, or cycloplegic refraction	Spherical equivalent refractive error
f_eye2_	mm	1000∙n/P_eye_	Back focal distance of the eye from ${\mathrm{pp}}_{\mathrm{eye}2}$
F	mm	pp_eye2_ + f_eye2_	From ant. corneal apex to back focal point

Abbreviations: Ant., anterior; D, dioptre; incl., including; post., posterior; pp., principal point.

Two parameters were especially important, namely, the total power of the eye P_eye_ and the axial power of the eye P_ax_, which is the power needed to emmetropise an eye having a certain AL. The difference between these powers may be considered as a definition of the refractive error:

(1) $$\mathrm{SE}=P_{\mathrm{ax}}-P_{\mathrm{eye}}$$

### Biometry scaling

It is sometimes useful to rescale biometric parameters to bring all eyes within a common framework. One easy way to do this is to divide all the physical distances inside the eye (e.g., intraocular distances, positions of cardinal points) by a reference—essentially expressing them as a percentage of this reference. In practice, there are two distances that are suitable as a reference: the AL or the distance F between the anterior corneal apex and the posterior focal point of the eye. Both references highlight different aspects of the eye, where AL stresses the differences in F and F the differences in AL. Since in emmetropic eyes both references are approximately equivalent, the AL reference was used here.

This relative scale can also be expanded to surface powers as follows. Starting, for example, from the anterior corneal radius of curvature r_ca_, the anterior surface power is given by

(2) $$P_{\mathrm{ca}}=1000∙\frac{n_{c}-n_{\mathrm{air}}}{r_{\mathrm{ca}}}$$

with all parameters defined in Table 1. Scaling radius of curvature r_ca_ by AL then gives

(3) $$P_{\mathrm{ca}}\left(\text{Scaled}\right)=\frac{n_{c}-n_{\mathrm{air}}}{r_{\mathrm{ca}}/\mathrm{AL}}=\frac{n_{c}-n_{\mathrm{air}}}{r_{\mathrm{ca}}}\mathrm{AL}$$

In other words, the surface powers scale through multiplication by AL. This principle also applies to the thick lens equation under isotropic scaling (i.e., all existing proportions remain the same):

(4) $$P_{c}\left(\text{Scaled}\right)=P_{\mathrm{ca}}\mathrm{AL}+P_{\mathrm{cp}}\mathrm{AL}-P_{\mathrm{ca}}P_{\mathrm{cp}}\mathrm{AL}∙\frac{\mathrm{LT}}{n_{c}}=P_{c}\mathrm{AL}$$

Since this relationship applies to all surface powers, the positions of the cardinal points must be scaled exactly the same way as the lens dimensions. Scaling a refractive optical system is therefore simply dividing all distances by the reference, while multiplying all power values by the same amount. Note that the reference AL has units of millimetres, making the scaled parameters unitless.

### Bigaussian analysis

As shown previously,^35^, ^36^ the leptokurtic and skewed distribution of the refractive error may be fitted by a bigaussian function of the following form:

(5) $$\text{Dist}=a_{1}∙\exp\left[-{\left(\frac{\mathrm{SE}-\mu_{1}}{\sigma_{1}}\right)}^{2}\right]+a_{2}∙\exp\left[-{\left(\frac{\mathrm{SE}-\mu_{2}}{\sigma_{2}}\right)}^{2}\right]$$

where a_i_ represents the amplitude of each Gaussian function, while μ_i_ and σ_i_ are the corresponding average and standard deviation values, respectively. These functions correspond to the Regulated and Dysregulated subpopulations.^35^ A generalised, higher‐dimensional version of this function may also be used to develop a similar model to express the relationships between the biometric parameters.^36^ Here the μ_i_ become two arrays of average values and the σ_i_ two covariance matrices. The fit algorithm is based on a k‐means algorithm to estimate the initial conditions, followed by maximum likelihood fit. To avoid the influence of local maxima, the algorithm was repeated 1000 times and the fit with the lowest Akaike Information Criterion, a measure for the goodness of fit, was used. To delineate the Regulated and Dysregulated subgroups, covariance ellipses with axes were derived using the eigenvectors of each parameter pair. These eigenvectors were multiplied by a factor ε to scale the ellipse size to match the sizes of the subgroups identified in the refractive distribution.

### Statistics

All data processing was performed using Matlab (v2020a, mathworks.com), MS Excel (v365, Microsoft.com) and SPSS (v25, ibm.com). Multivariate bigaussian fits to a selection of biometric parameters were performed using a standard Matlab function (‘fitgmdist’). Covariance ellipses were subsequently determined for each pair of biometric parameters using the associated eigenvectors as the long and short elliptical axes.

All statistical tests were performed at a significance level of *p* < 0.05. If necessary, this was modified by a Bonferroni correction to minimise the risk of alpha inflation. Note that many of the parameters considered were not normally distributed, which typically requires the use of non‐parametric statistics during the data analysis. However, since this requirement does not apply to large datasets, even in cases of extreme non‐normality,^44^ standard parametric statistics were used instead.

## RESULTS

This section features the most important tables and figures. Additional analyses and alternative visualisations such as figures, animations and tables can be found in Appendix S1.

### Biometric variations

#### Emmetropia

The study cohort contained 979 emmetropic eyes, for which the average biometric values are provided in Table 2. Emmetropia was found in eyes with an AL ranging between 20.86 and 25.62 mm, a difference of 4.76 mm that corresponds with an axial power P_ax_ range of 14.82 D (from 56.48 D to 71.30 D). As can be expected in emmetropic eyes, the power of the whole eye, P_eye_, showed a range close to that of P_ax_, that is, from 56.62 D to 71.63 D. Meanwhile, the corneal power P_c_ showed a range of 8.48 D, while the lens power P_lb_ had a larger range of 12.34 D.

**TABLE 2:** Ocular biometry for the emmetropic eyes and the entire cohort (mean ± standard deviation [SD] [range]).

Parameter	Unit	Emmetropic eyes (*N* = 979)	All (*N* = 2000)	*t*‐test (*p* ^a^)
**Age**	years	48.9 ± 5.3 [40, 64]	49.2 ± 5.7 [39, 64]	0.15
**SE**	D	0.08 ± 0.32 [−0.50, 0.50]	0.02 ± 1.52 [−14.75, 8.38]	0.27
**CCT**	mm	0.53 ± 0.03 [0.43, 0.67]	0.53 ± 0.03 [0.42, 0.67]	0.46
**AD**	mm	2.66 ± 0.30 [1.80, 3.76]	2.64 ± 0.32 [1.70, 3.95]	0.33
**LT**	mm	4.24 ± 0.27 [3.39, 5.26]	4.25 ± 0.29 [3.32, 5.26]	0.55
**VD**	mm	15.70 ± 0.71 [13.44, 18.37]	15.72 ± 0.85 [12.86, 22.26]	0.53
**AL**	mm	23.12 ± 0.74 [20.86, 25.62]	23.14 ± 0.90 [20.27, 30.55]	0.68
**P**_**c**_	D	41.97 ± 1.41 [37.71, 46.20]	42.02 ± 1.47 [36.25, 47.61]	0.47
**P**_**lb**_	D	25.98 ± 1.85 [19.98, 32.32]	26.00 ± 1.88 [19.53, 34.50]	0.80
**P**_**eye**_	D	63.37 ± 2.24 [56.48, 71.30]	63.43 ± 2.25 [56.47, 71.65]	0.50
**P**_**ax**_	D	63.52 ± 2.26 [56.62, 71.63]	63.50 ± 2.63 [47.96, 73.87]	0.85

*Note*: Parameters are defined in Table 1; full version, split up by sex, is available in Table S1.D, dioptres.

^a^ Unpaired *t*‐test comparing emmetropic eyes with the entire cohort; *p* < 0.05/11 = 0.0045 (Bonferroni correction) is significant (in bold).

#### Ametropia

As in most previous studies, the distribution of refractive errors in this cohort was leptokurtic and skewed, and can accurately be described by a bigaussian function^35^, ^36^ (Figure S1). The prevalence of myopia in this cohort was 19.8%.

No significant differences were found in the average parameter values over the entire cohort compared with the corresponding values of emmetropic eyes, including the SE refractive error (Table 2). Over the entire cohort, SE ranged between −14.75 D and +8.38 D, a difference of 23.13 D. The AL had a range of 10.28 mm, from 20.27 mm to 30.55 mm, corresponding to an axial power P_ax_ range of 25.91 D (from 47.96 D to 73.87 D). This P_ax_ range roughly agrees with the range in SE. The whole eye power P_eye_, on the other hand, had a far smaller range of 15.17 D, similar to that of the emmetropic eyes. Both observations agree that refractive error is mostly axial in nature. Finally, the corneal and lens powers had ranges of 11.36 D and 14.97 D, respectively, which in both cases weFre larger than that found in emmetropic eyes.

### Correlations

#### Emmetropia

To reach emmetropia, a clear inverse relationship should exist between AL and the refractive powers of the cornea, P_c_, and lens, P_lb_ (or the whole eye P_eye_). These relationships are indeed present, as clearly indicated by the high and significant correlation values (Table 3). These correlations were particularly high between P_eye_ and AL (*r* = −0.978, *p* < 0.001) and P_eye_ and P_ax_ (*r* = 0.989, *p* < 0.001), as can be expected in emmetropic eyes. Due to the narrow range of refractive error SE, the correlations between biometric parameters and SE were all either weak or non‐significant, also as expected.

**TABLE 3:** Overview of Pearson correlations and *p*‐values between the biometric parameters for the emmetropic eyes (*N* = 979, blue above diagonal) and the entire cohort (*N* = 2000, orange below diagonal). Darker colours correspond with stronger correlations.

	SE	AD	LT	VCD	AL	P_c_	P_l_	P_eye_	P_ax_
SE	–	−0.119	0.016^a^	−0.088^a^	−0.124	−0.084^a^	0.021^a^	−0.022^a^	0.119
AD	−0.313	–	−0.541	0.262	0.442	−0.060^a^	−0.434	−0.403	−0.416
LT	0.191	−0.576	–	−0.345	−0.170	0.019^a^	0.310	0.221	0.221
VCD	−0.552	0.345	−0.362	–	0.930	−0.775	−0.734	−0.943	−0.950
AL	−0.570	0.491	−0.225	0.951	–	−0.759	−0.756	−0.978	−0.989
P_c_	−0.111	−0.008^a^	−0.023^a^	−0.588	−0.569	–	0.222	0.719	0.704
P_lb_	−0.085	−0.367	0.273	−0.525	−0.537	0.174	–	0.832	0.828
P_eye_	−0.098	−0.337	0.181	−0.723	−0.744	0.705	0.815	–	0.990
P_ax_	0.526	−0.475	0.268	−0.958	−0.987	0.539	0.640	0.794	–

*Note*: Parameters defined in Table 1. Full version of the table available in Table S2.

^a^ Non‐significant correlation with *p* > 0.05/11 = 0.0045 (Bonferroni correction).

To some degree, the intraocular distances for emmetropic eyes scale up with AL as it is correlated with AD (*r* = 0.442, *p* < 0.001) and VCD (*r* = 0.930, *p* < 0.001). Therefore, longer emmetropic eyes tended to have deeper anterior and vitreous chambers. Meanwhile, only a weak inverse correlation was seen with LT (−0.170, *p* < 0.001).

**FIGURE 1:**
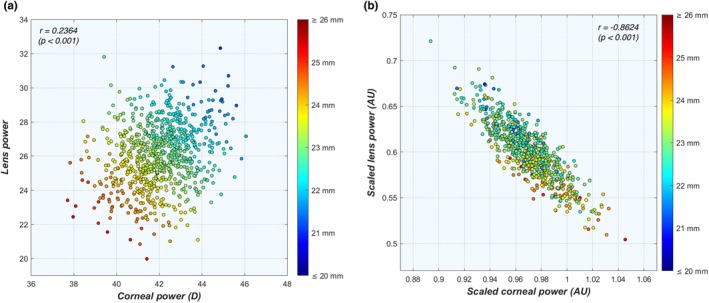
Scatter plots of lens power P_l_ as a function of corneal power P_c_ in emmetropic eyes using (a) the original parameter values and (b) values scaled according to AL. Colour gradient represents AL. AL, axial length. AU, arbitrary units.

Although the cornea and lens are complementary in providing the adequate refractive power to obtain a sharp retinal image, only a weak correlation was found between these parameters (*r* = 0.222, *p* < 0.001). While this may appear surprising for an emmetropic cohort, it is important to remember the large 4.76 mm range in emmetropic ALs (or 14.82 D in axial power) that obfuscates this correlation. Indeed, a scatter plot of P_c_ and P_lb_ shows no mutual relationship (Figure 1a), but as both powers are correlated with AL, the point cloud does show a clear relationship with AL, as indicated by the colour gradient. If one were to scale P_c_ and P_lb_ according to AL using Equations (2–4), a considerably stronger correlation emerges (*r* = −0.862, *p* < 0.001; Figure 1b), confirming the importance of AL in their relationship.

#### Ametropia

Overall, the correlations between the biometric parameters considered over the entire cohort show similar patterns to those of emmetropic eyes, with a few notable exceptions. Due to the large range in refractive error SE, significant correlations appear between SE and the intraocular distances AL (*r* = −0.570, *p* < 0.001), VCD (*r* = −0.552, *p* < 0.001) and AD (*r* = −0.313, *p* < 0.001). Meanwhile, only weak inverse correlations were seen between SE and the powers of the cornea, lens and the whole eye (*r* = −0.111, *p* < 0.001; *r* = −0.085, *p* < 0.001; *r* = −0.098, *p* < 0.001, respectively), again confirming the predominantly axial origins of refractive error. The fact that these latter correlations are inverse is noteworthy, as this means that myopic eyes would have higher refractive corneal and lens powers than emmetropic eyes and the reverse for hypermetropic eyes, which in both cases increases the level of ametropia. This observation was also reported by Sorsby.^17^, ^21^ Another difference with emmetropic eyes is the lower correlation between the power of the eye P_eye_ and the axial power P_ax_, decreasing from *r* = 0.990 in emmetropic eyes to *r* = 0.794 for the entire population due to the influence of the ametropic eyes.

### Overlap of distributions

**FIGURE 2:**
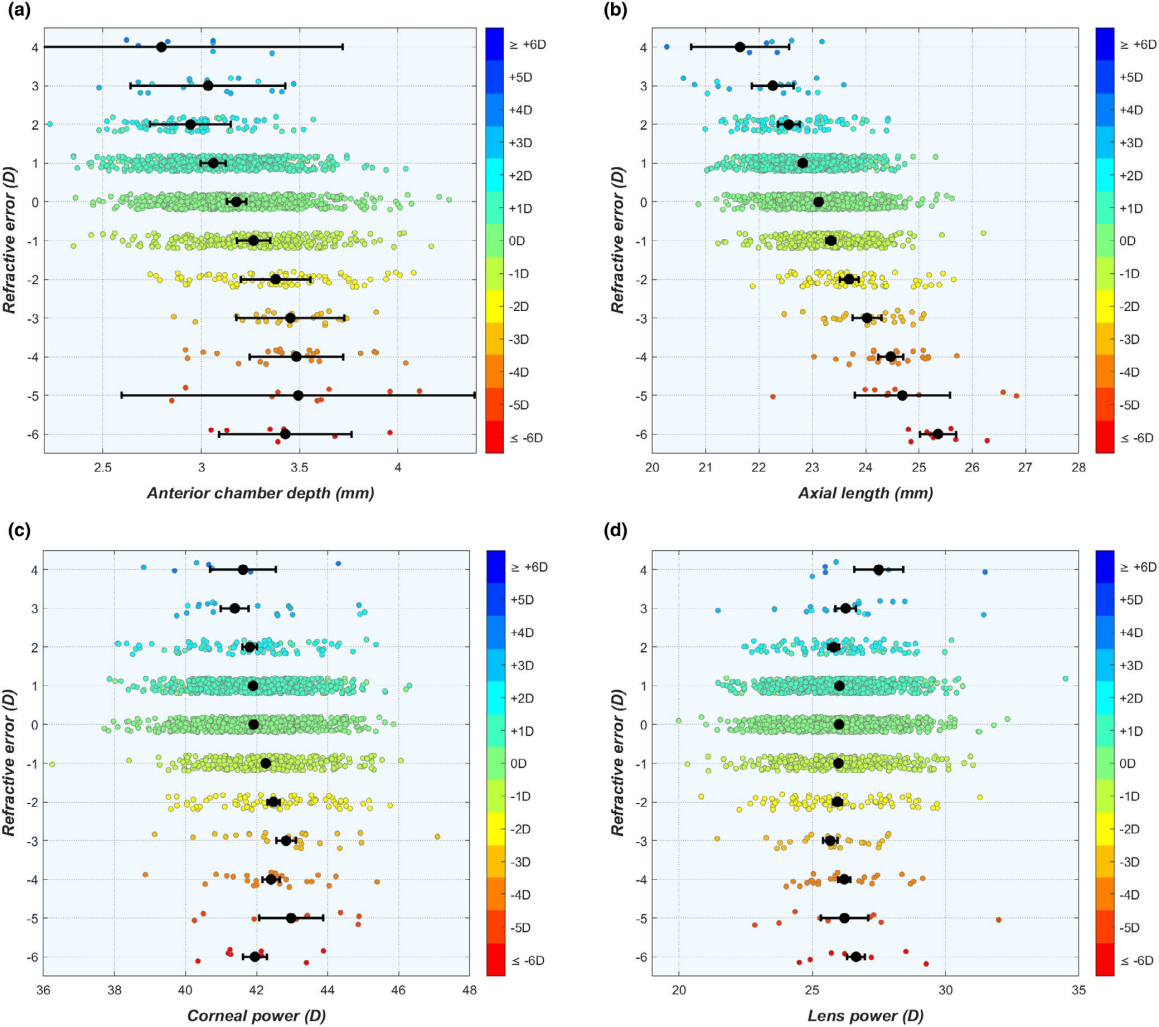
While the average values of many biometric parameters are significantly different between refractive groups, there is still considerable overlap. This is illustrated for (a) corneal power P_c_, (b) lens power P_l_, (c) anterior chamber depth ACD_tot_ and (d) AL. Black marks indicate the average for each refractive bin and error bars the 95% confidence interval of the standard error. Small vertical variations in the position of each point were added to illustrate the number of individual eyes in each group. ACD, aqueous depth; AL, axial length.

After dividing the data according to the SE refractive error in 1 D bins, clear and significant changes were seen in the average values of anterior chamber depth ACD_tot_ and AL that increased with increasing levels of myopia (Figure 2a,b; numerical values in Table S3). Meanwhile, the difference in corneal power P_c_ between the refractive groups was less pronounced, albeit significant, with higher P_c_ values for higher levels of myopia (Figure 2c). For lens power P_l_, significant changes were present only for the most myopic and hypermetropic refractive errors, while the lens power was essentially constant in the range of ±3 D (Figure 2d). Simultaneously, it can be seen that the ranges of the parameters for the refractive groups strongly overlap for all four biometric parameters, especially for refractive errors near emmetropia. This matches observations by Berg^13^ and Sorsby et al.^17^ and can be visualised by comparing scatter plots of the emmetropic and ametropic eyes side by side (see Figures S2 and S3). The only graph where the values of the different refractive groups do not overlap is for the whole eye power P_eye_, as a function of the axial power P_ax_ (Figure S3d), which is somewhat trivial as it is essentially the definition of refractive error used in Equation (1).

**FIGURE 3:**
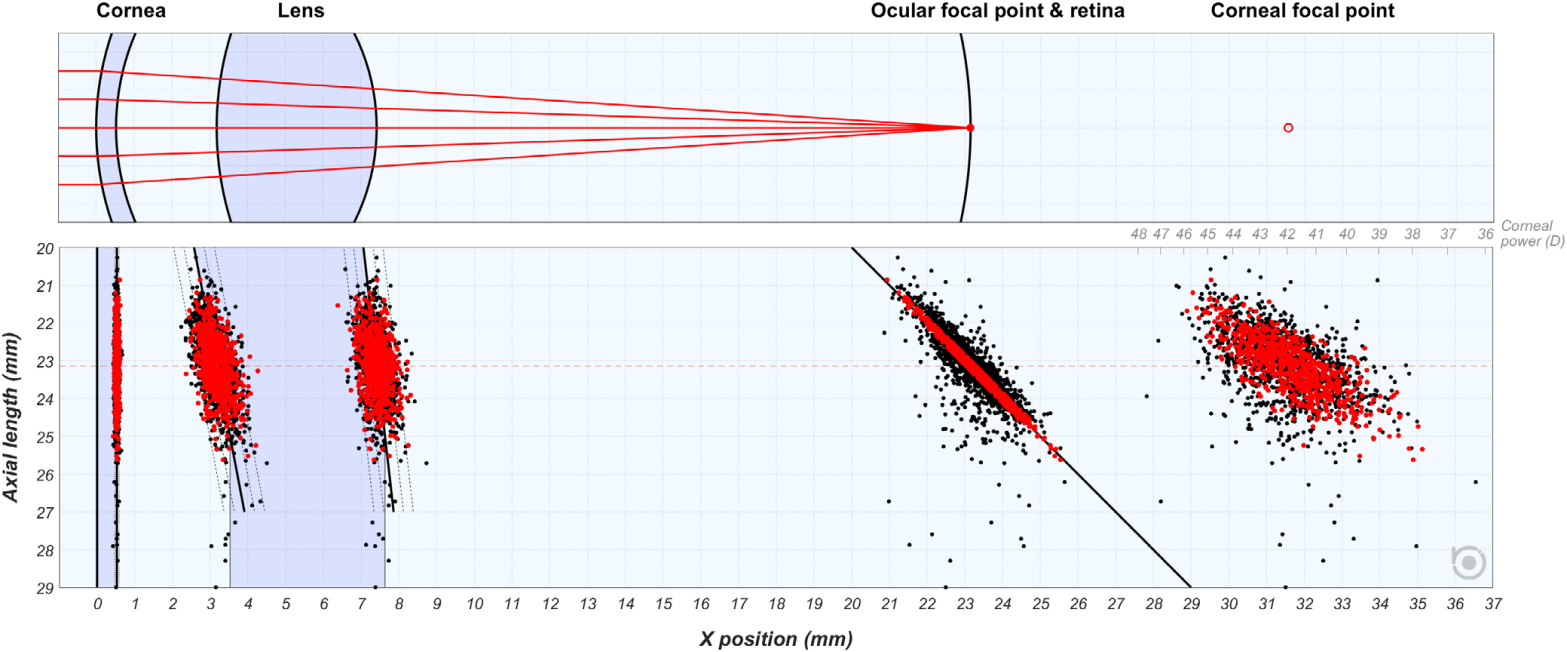
Top: Layout of the average eye based on the entire population, with the lens radii of curvature estimated using Royston's equations^45^ (not used in calculations, only for visualisation). Bottom: Positions of the refractive surfaces and ocular and corneal focal points for all eyes (black) and emmetropic eyes (red), sorted based on axial length. The anterior cornea is located at *x* = 0 mm, and the diagonal line at 20–29 mm represents the position of the retina.

Another way to demonstrate the overlap between refractive groups is by marking the positions of the refracting surfaces and focal points of the eye as a function of the AL (Figure 3), which also visualises the correlations reported in the previous section, such as deeper anterior chambers and flatter corneas for longer eyes. The positions of the lens surfaces, corresponding with the ACD_tot_ and the anterior segment length ASL, follow these linear regressions as a function of the AL in all eyes between 20 and 27 mm:

(6) $$\begin{array}{c} {\mathrm{ACD}}_{\mathrm{tot}}=0.1927∙\mathrm{AL}-1.2859\;\left(r^{2}=0.264\right)\\ \mathrm{ASL}\;=0.1126∙\mathrm{AL}+4.826\;\left(r^{2}=0.116\right) \end{array}$$

These regressions are shown in Figure 3 as black diagonal lines, along with the standard deviation of the residuals (0.27 and 0.26 mm for ACD_tot_ and ASL, respectively—dotted lines). The cut‐off of 27 mm was chosen based on the observation that up to this value the eyes still follow regressions (6), while in eyes longer than 27 mm any correlation with AL seemed to be absent.

Based on the slopes of these regressions, the anterior lens surface is positioned 1.16 mm deeper in eyes with an AL of 27 mm, compared with 21 mm, while the posterior surface is deeper by only 0.68 mm between these two extremes. This difference corresponds with a lens that is thinner by 0.48 mm in the longer eyes. Both deeper positions and thinner lenses are typically associated with the lower (effective) lens powers needed in longer eyes.

**FIGURE 4:**
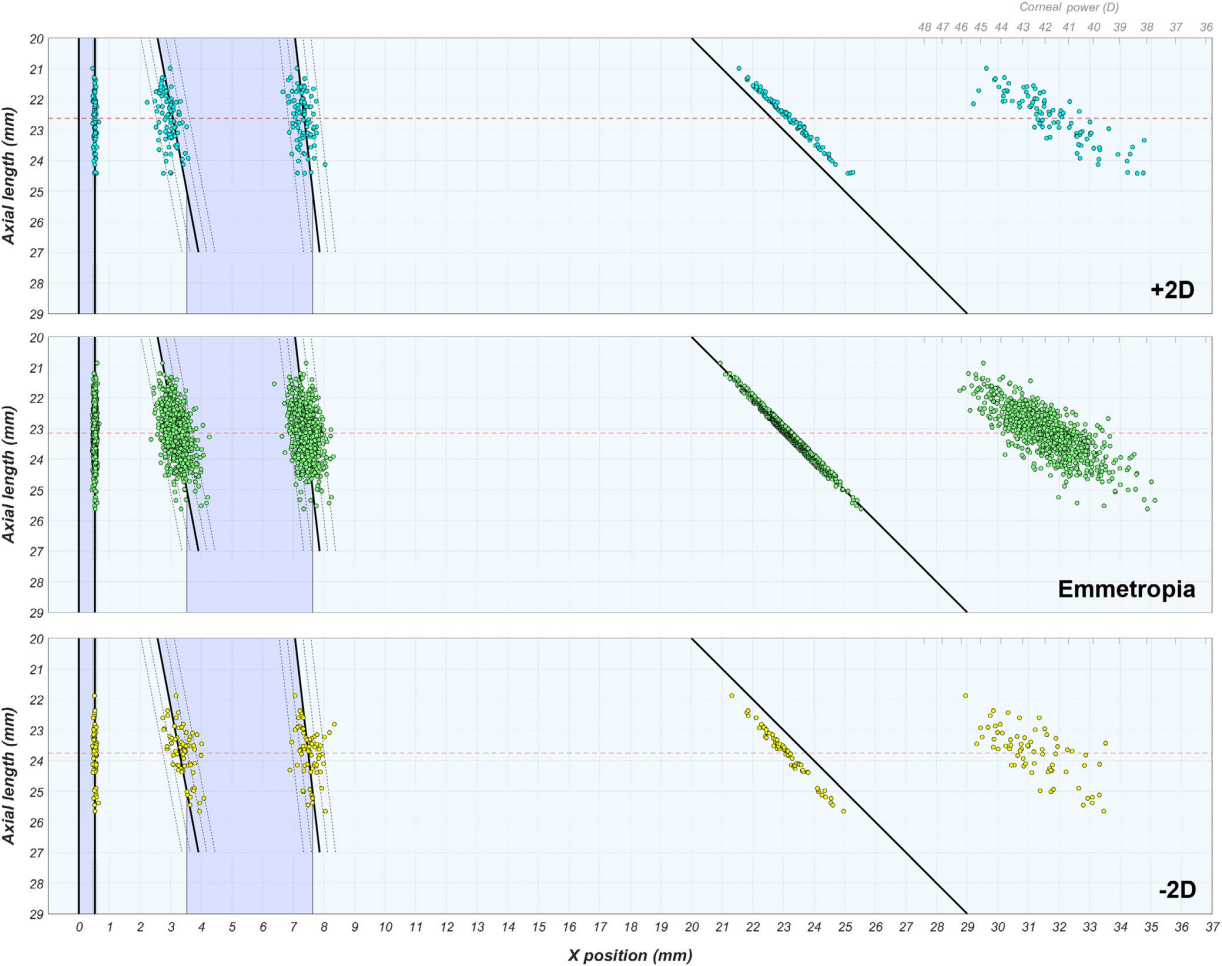
Positions of the refractive surfaces and the ocular and corneal focal points, sorted according to axial length, for three levels of refractive error. The anterior cornea is located at *x* = 0 mm. The red dashed line represents the average axial length and the diagonal line the retina. For the full range, see the animation in Figure S4.

Comparing the emmetropic eyes (in red) and the entire cohort (in black), the point clouds of the lens surface positions and the corneal focal point overlap almost completely. Meanwhile, both groups have only a minimal overlap for the point cloud of the ocular focal point, which lies on the retina for the emmetropic eyes, and either in front or behind the retina for myopic and hypermetropic eyes, respectively. When dividing the entire cohort into refractive groups (Figures 4 and S4), again there is an overlap between successive groups, but also each refractive group down to –6 D seems to be bound by the same regression lines and confidence intervals. The point cloud representing the corneal focal point position gradually moves left (Figure S4), again confirming that the average corneal power is higher in longer eyes.^17^, ^21^

### Uniqueness

The density of the scatter plots in Figures 3 and 4 may give the impression that there should be many eyes with closely resembling ocular biometry. To test the uniqueness of each eye, six biometric parameters for each eye (SE, P_c_, P_lb_, ACD_tot_, LT and AL) were compared with those of all others, within certain levels of tolerance based on the repeatability of the biometric devices used. Based on the literature,^46^, ^47^ the repeatability levels chosen were 0.25 D, 0.12 D, 0.25 D, 0.009 mm, 0.015 mm and 0.019 mm, respectively. Using these tolerances, only three pairs of emmetropic eyes could be found in the cohort of 2000 eyes within the limits of agreement (i.e., 1.96 times the repeatability), making these the only pairs indistinguishable with the biometric equipment used. Hence, ocular biometry seems to be fairly unique, even in such a large cohort.

### Regressions

**FIGURE 5:**
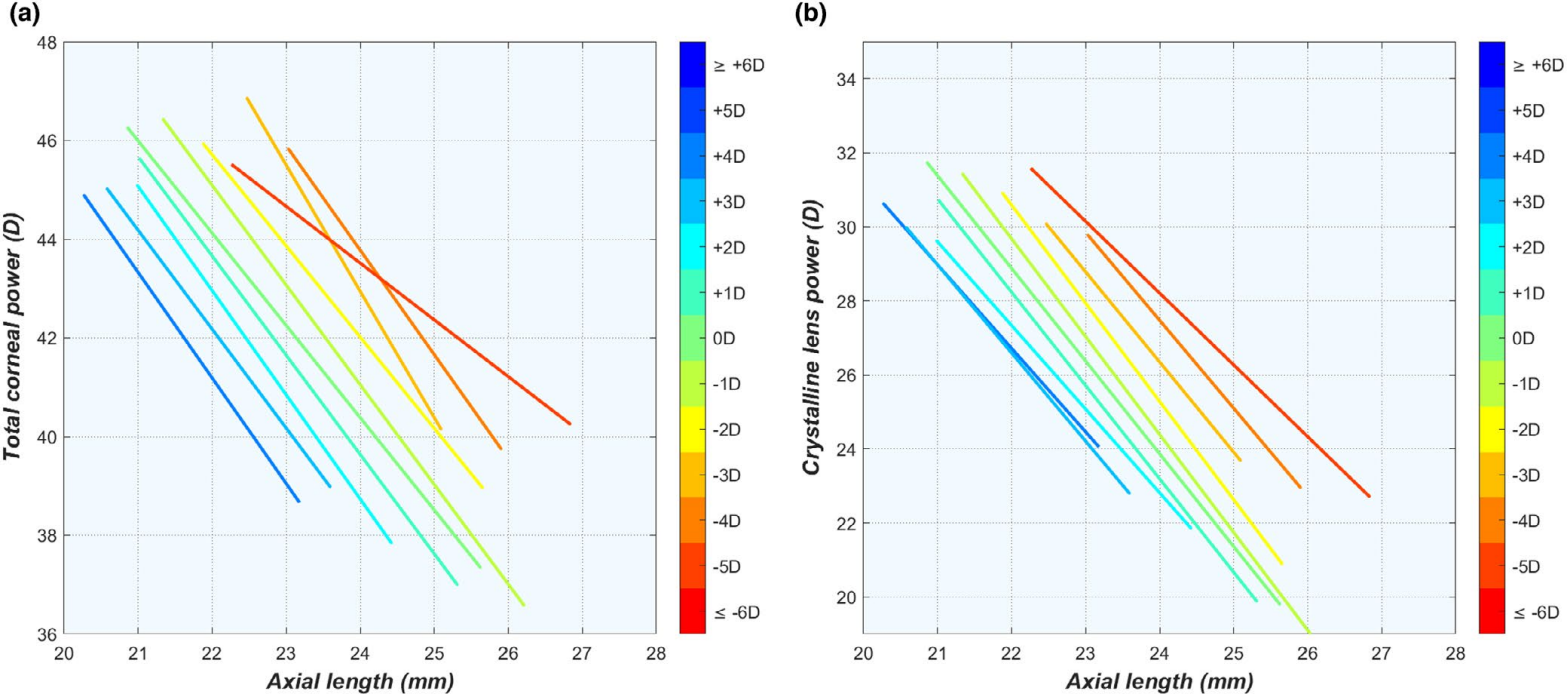
Linear regressions of the ocular biometry parameters as a function of axial length considered for the refractive groups. (a) Corneal power and (b) lens power. Other examples in Figure S5.

The point clouds in Figures 3 and 4 suggest that the slopes of the linear regressions of the biometric parameters, as a function of AL for each refractive group, may be very similar. This is indeed observed for the correlations between AL and corneal power P_c_, lens power P_l_ or ACD_tot_, all of which are essential in creating the refractive balance in the eye (Figure 5, see also Figure S5 and Table S4).

### Bigaussian analysis

#### Refractive distribution

**FIGURE 6:**
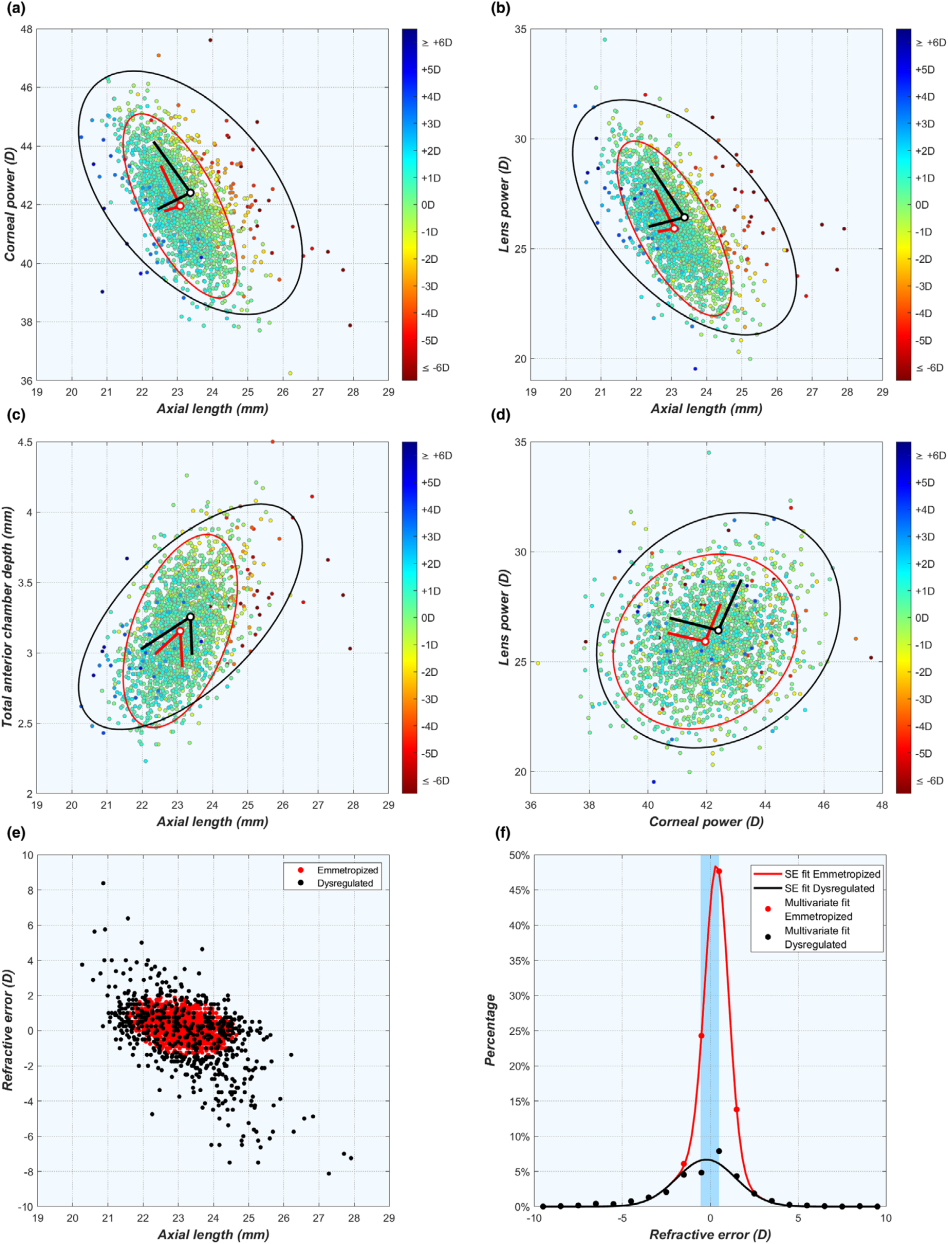
(a–d) Four examples of multivariate bigaussian fits of the ocular biometry, dividing the population into the Regulated (red) and Dysregulated (black) subgroups (more examples available in Figure S6). Ellipses represent the 97.62% confidence region for each subgroup as explained in the text. Vectors indicate the eigenvectors for each subgroup. Although these eigenvectors are perpendicular, this may not be apparent due to scale differences on the horizontal and vertical axes. (e) Scatter plot of Regulated and Dysregulated eyes. (f) Bigaussian fit of the refractive distribution.

As established previously, the refractive error and biometric parameters may be represented using bigaussian functions. Fitting the refractive distribution between ±10 D (*N* = 1994) in 1 D intervals with such a function yields

(7) $$\text{Dist}=0.423∙\exp\left[-{\left(\frac{\mathrm{SE}-0.372}{0.935}\right)}^{2}\right]+0.0668∙\exp\left[-{\left(\frac{\mathrm{SE}-0.226}{2.369}\right)}^{2}\right]$$

with *r* ^2^ = 0.99. These terms correspond to the Regulated and the Dysregulated subpopulations, with relative weights of 71.4% and 28.6%, respectively. Looking only at the refractive error, both populations overlapped in the range between −1.5 D and +2.5 D, and individual eyes in that range cannot be assigned to either subgroup based on the refractive information alone (Figure 6f).

#### Biometry

To discriminate between Regulated and Dysregulated eyes better, the refractive analysis was expanded as a sum of two multivariate Gaussian distributions, fitted over the combined data of the refractive error SE, total corneal power P_c_, lens power P_lb_, total anterior chamber depth ACD_tot_ and AL, again taken from the 1994 eyes with an SE refractive error between −10 D and +10 D. The details of the best fit are listed in Table S5. The results of the best fit are shown in Figure 6 for various combinations of biometric parameters, where the red and black lines represent the covariance ellipses of the Regulated and Dysregulated eyes, respectively.

The covariance ellipses may now be used to discriminate between the two subpopulations, by identifying all eyes with biometric values simultaneously inside the red ellipses of all pairs of parameters as Regulated. Those with at least one point outside the red ellipse were identified as Dysregulated. Using this definition, the eigenvectors of each parameter pair may be multiplied by a factor *ε* so that the balance between both subpopulations closely matches that of the refractive error distribution in Equation (7). This was the case for *ε* = 2.26·σ, with 70.3% Regulated and 29.7% Dysregulated. This ε value corresponds with a confidence interval containing 97.62% of points for each subpopulation.

Note that the Regulated subgroup cannot be equated with the subgroup of emmetropic eyes as the former contains 1401 eyes with refractive errors ranging between 1.46 D and +2.20 D, while the latter includes 979 eyes with |SE| ≤ 0.5 D. Further, 41.97% of the Regulated subgroup were not emmetropic by that definition (see blue band in Figure 6f). Meanwhile, 83.04% of the emmetropic eyes fell in the Regulated group, while 16.96% of emmetropic eyes were Dysregulated (Figure 6e,f). In this latter group, a deviation in one or more biometric parameters was compensated simultaneously by the other parameters, allowing these ‘lucky’ Dysregulated eyes to still emmetropise.

### Emmetropic variations

In this cohort, emmetropia occurred in ALs ranging between 20.86 and 25.62 mm, which means that the powers of the cornea and lens must also vary considerably in a complementary fashion to provide a sharp retinal image for each of these ALs. Theoretically, there could be two ways in which P_c_ and P_l_ scale according to AL: either through a simultaneous proportional downscaling of both powers or a balancing process between both so that their combined power decreases proportionally with AL, but not necessarily with their individual powers.

**FIGURE 7:**
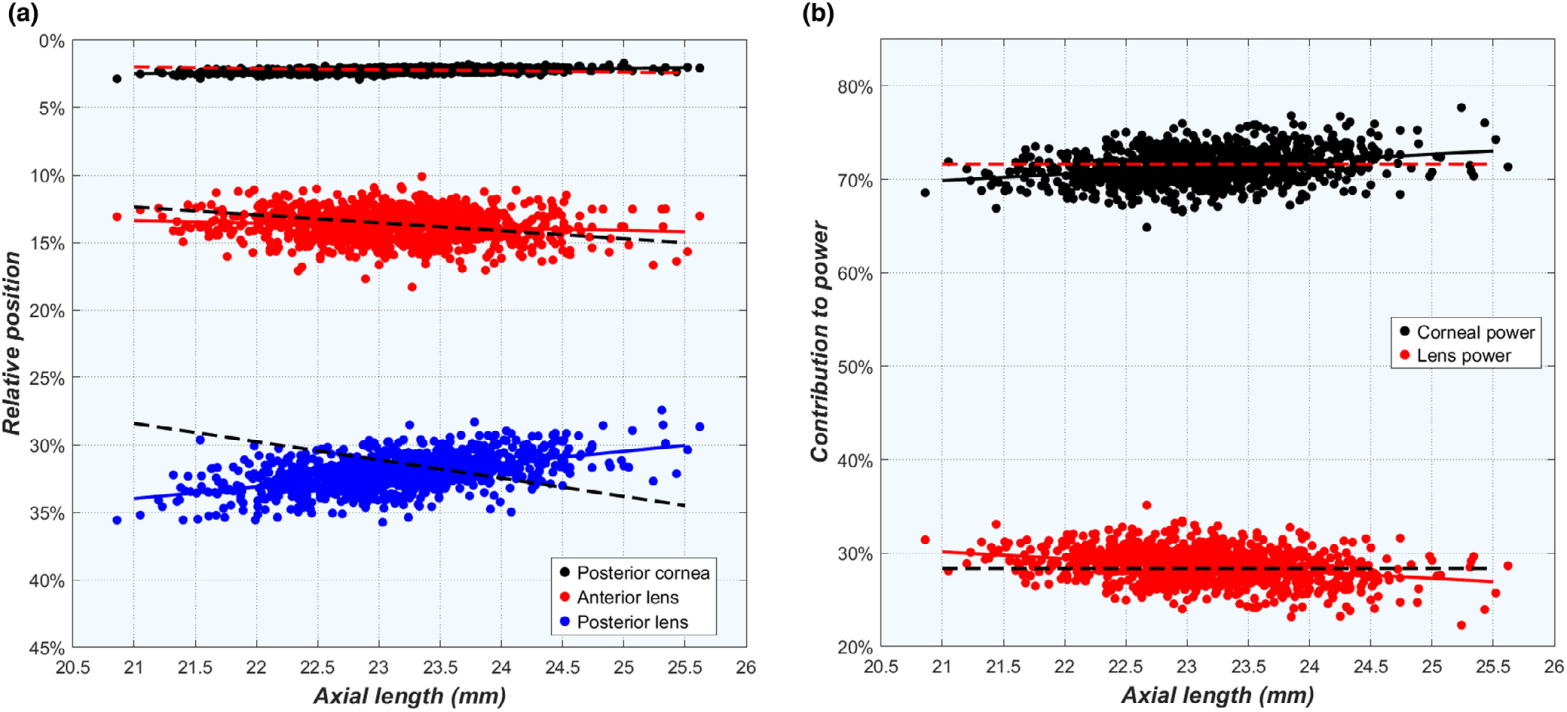
(a) Position of the refractive surfaces scaled to AL in all emmetropic eyes, with the anterior cornea at 0%. (b) Relative contributions to the total refractive power of the eye of the cornea (P_c_/P_eye_) and the lens (P_l_/P_eye_), both calculated at the ocular principal planes. Dashed lines indicate the reference in the case of proportional scaling of a 23.5‐mm eye, while retaining the same refractive contributions by the cornea and lens. AL, axial length.

To explore this further, the positions of the four refractive surfaces in the eye were scaled to AL as a percentage (Figure 7a). Using this frame of reference, the position of the posterior cornea was 0.40% closer to the anterior cornea in an emmetropic eye that is 26 mm long compared with a 21 mm eye, which, given the lack of correlation of corneal thickness CCT to any other parameter, is of minimal importance. Meanwhile, AD was correlated with AL, so the relative position of the anterior lens surface was fairly stable with a 0.8% deeper position in the 26 mm emmetropic eye. Finally, the relative position of the posterior lens surface showed a much larger difference of 3.94%, close to the cornea at 26 mm. These latter two observations indicate that the relative LT is considerably smaller in long eyes, mostly originating from the posterior segment.

Looking at the ocular refractive powers, the corneal and lens powers at the ocular principal plane were 70% and 30% of the total ocular power, respectively, which decreases with AL at a rate of –1.66 D/mm AL (*r* ^2^ = 0.579) and –1.29 D/mm AL (*r* ^2^ = 0.606), respectively. This causes the relative contribution of the cornea to the overall refractive power to be 3.2% higher in a 26 mm emmetropic eye than for an eye that is 21 mm long, while the opposite effect is seen for the contribution of lens power (Figure 7b). This, along with the results shown in Figure 1, confirms that the balance between P_c_ and P_l_ is essential to accomplish emmetropia, rather than proportional downscaling. Consequently, short and long emmetropic eyes have a slightly different refractive balance between their components.

An animation of the biometric and refractive changes in emmetropic eyes, along with the linear regressions needed to produce this refraction, is available in Figure S7.

## DISCUSSION

Using data from the ShECS, this work revisited the classic results of Sorsby et al.^17^, ^18^, ^19^, ^20^, ^21^ and reaffirmed the importance of their contributions to understanding of how the eye achieves and maintains emmetropia. The observed correlations between the ocular components and unique biometric profiles of each eye underscore the complex interplay between AL and the refractive powers of the cornea and lens, even within a seemingly homogeneous population.

The observations also confirm the bigaussian nature of ocular biometry, with Regulated and Dysregulated eyes being two distinct subpopulations, while a deeper analysis of the biometric overlap of both subgroups near emmetropia offers new insights into the biometric characteristics that differentiate these groups.

The following sections discuss these aspects in more detail.

### Defining emmetropia

As Steiger^7^ noted, classification of refractive errors is based purely on dioptric consideration rather than biological dimensions. This is especially true within the range of ±3 D, where the biometric values show near‐complete overlap between refractive groups (Figure 3), as was also reported by Berg^13^ and Sorsby.^17^ Consequently, the only accurate way to delineate emmetropia is by definitions based on refractive error, such as |SE| ≤ 0.5 D or |SE| < 0.5 D. However, as refractive error is not really a feature of the eye as such, but rather the imbalance between the dioptric elements and the AL, it makes sense to look also at the biometric limits of emmetropia.

Many clinicians seem to be under the impression that emmetropia in adults is limited to ALs between 23.5 and 24.0 mm. However, the AL range reported here spans the far wider range of 20.86–25.62 mm. Apart from being about 0.3 mm shorter than the (mostly European) findings in the literature and the large variety of methods used to obtain the values, they match roughly the reported overall range of about 21.0–26.5 mm (Table 4). Note that clinically, the extremes of this range are typically associated with moderate levels of hypermetropia and myopia, respectively.

**TABLE 4:** Emmetropic axial lengths for adults in the literature using various measurement methods.

Author(s) (year)	Refractive range	Method	*N*	Mean ± SD [min, max]
Tron (1934)^48^,^a^	−0.5 ≤ SE ≤ 0.5	Calculated	89	24.05 ± 0.99 [22.19, 27.30]
Strömberg (1936)^12^	−0.5 ≤ SE < 0.5	Calculated	754	24.48 ± 0.51 [22.75, 26.25]
Stenström (1946)^9^,^a^	−1.0 < SE < 1.0	X‐ray	651	23.95 ± 0.71 [21.75, 26.25]
Deller et al. (1947)^49^	−0.5 < SE < 0.5	X‐ray	19	22.8 ± 1.2 [21.0, 25.0]
Otsuka and Kanefuji (1951)^50^,^a^	−0.5 < SE < 0.5^b^	X‐ray	86	23.59 ± 1.00 [20.5, 26.0]
Sorsby et al. (1957)^17^	−0.5 ≤ SE ≤ 0.5	Calculated	63	24.16 ± 0.87 [22.19, 26.30]
François and Goes (1977)^30^	−1.0 < SE < 1.0^b^	Ultrasound	100	23.37 ± 0.75 [21.50, 24.94]
Larsen (1979)^51^	−0.5 < SE < 0.5^b^	Ultrasound	40	23.82 ± 0.81 [22.15, 25.74]
Kuzmanović Elabjer (2007)^52^,^c^	−0.5 < SE < 0.5^b^	Ultrasound	1000	23.45 ± 0.74 [21.04, 26.40]
Rozema et al. (2014)^36^,^a^	−0.5 ≤ SE ≤ 0.5	Optical	388	23.41 ± 0.74 [21.36, 26.16]
Bikbov et al. (2019)^53^,^a^	−0.5 ≤ SE ≤ 0.5	Optical	768	23.35 ± 0.83 [21.12, 27.34]
Current	−0.5 ≤ SE ≤ 0.5	Optical	979	23.12 ± 0.74 [20.86, 25.62]

Abbreviations: SD, standard deviation; SE, spherical equivalent.

^a^ Estimated from the available data.

^b^ Not specified whether boundaries were included.

^c^ Estimated by combining values for males and females.

To emmetropise such widely differing eye sizes, it is essential that the refracting elements of the eye, that is, the cornea and lens, adjust their powers accordingly (Figure 1b). But rather than each element adjusting its power independently according to the scale of the eye, the powers of both elements appear to be coordinated so that together they produce the necessary power required for emmetropia. Oddly enough, longer emmetropic eyes seem to have corneas that are relatively more powerful and lenses that are relatively less powerful (Figure 7). Moreover, as indicated by the scattered data in the same figure, the relationships between the ocular elements are far more complicated than can be described by simple linear regressions (e.g., Figure S7). Combining these observations with the (near) uniqueness of ocular biometry (as shown in the section entitled uniqueness within the results), it is fair to say that there are as many ways to be emmetropic as there are emmetropic eyes (and perhaps even more ways to be ametropic).

### Defining refractive errors

The wide variation in AL observed in emmetropic eyes is also seen in other refractive groups (Figure 4). Here too, the refracting elements have adjusted themselves accordingly, although in ametropia the eyes may miss the mark. Even so, the correlations and regression slopes of the parameters remain very similar across refractive groups (Table 4, Figure 5). These observations, along with the considerable overlaps in Figure 2, demonstrate that the interactions between the ocular dimensions will frustrate any attempt to understand accurately the origin of the refractive error based on the ocular biometry, unless all parameters or at least the AL, corneal power P_c_,^54^ Bennett lens power^40^, ^41^ P_lb_ and anterior chamber depth ACD_tot_ are considered. One could also consider the lens power in the corneal plane, thus eliminating the need for ACD_tot_. However, this option may have some limitations as it removes all information regarding lens position, and it would lead to considerably lower values that could potentially lead to confusion. Alternatively, one could report the amounts of ocular elongation that are either compensated or uncompensated by the refractive elements as a means to track normal and myopic refractive development.^31^, ^55^

Until recently, most studies of myopia development or myopia control in children only presented changes in cycloplegic refractive error as the parameter of interest and an assessment of the effectiveness of the myopia control method being considered. While this form of reporting is certainly understandable, it does not provide any information about how the eye is changing in response to the treatment. Most likely, axial growth slows down, but an alternative explanation could be that the crystalline lens is somehow able to lose power to (partially) compensate for axial growth. Nowadays, this situation has improved as most studies of myopia report AL alongside the refractive error, demonstrating that myopia control measures do indeed slow down axial growth considerably.^56^, ^57^ However, without insights into the changes in the corneal or lens powers, essential information remains hidden, as evidenced by recent studies that atropine drops not only slow down axial growth but also reduce the power of the crystalline lens^58^, ^59^ (although an investigation using the less sensitive Bennett–Rabbetts equation found no change^60^). These observations reiterate the need for a complete set of biometric values to understand the sources of ametropia and monitor progression.

Traditionally, there has been a distinction between axial myopia (defined as ‘A myopic refractive state primarily resulting from a greater than normal axial length’^61^) and refractive myopia (‘A myopic refractive state that can be attributed to changes in the structure or location of the image forming structures of the eye, i.e., the cornea and lens’^61^). However, as can be seen in Figures 2, 3, 4, real‐world myopia does not fit either of these definitions but rather is a combination of the contributions by the different ocular components that interact in the process of emmetropisation. Although this ‘holistic’ approach to determine the origins of refractive errors is not yet prevalent in the literature, several commercial systems have adopted this approach, such as the Gullstrand Refractive Analysis System (GRAS; integrated in the Oculus Myopia Master, oculus.de) and the mEYE map^62^ (Ocumetra, ocumetra.com). Both systems present the corneal power, lens power at the corneal plane and axial power of the eye as differences from a predefined reference (considering age, sex and ethnicity^63^) to show the refractive balance of the eye. Overall, it is likely that this more suitable holistic approach will receive more widespread adoption in the near future for myopia management, but perhaps also to identify eyes at risk of developing unexpected refractive outcomes after cataract surgery.^64^

Another important aspect is that previous reports have sometimes considered eyes with an SE refractive error ≤–6 D or an AL longer than 26 mm as having pathological myopia, referring to the increased risk of developing structural changes in the posterior segment of the eye.^65^ However, as can be seen in Figure 2b, these definitions refer to a different subgroup of eyes. Hence, it may be better not to link pathological myopia to a specific AL or refractive error threshold, but rather to pathological phenotypes, as suggested in the recent paper by Flitcroft et al.^61^

### Estimating biometric parameters

As clinicians and researchers are becoming increasingly aware of the importance of having a complete set of ocular biometry parameters in the analysis of myopic growth, others have developed methods to estimate values for missing parameters using regressions or machine learning methods, for example, to estimate the risk of future myopia development using growth curves.^66^, ^67^ These methods may be clinically useful in cases when data cannot be obtained due to time constraints or missing equipment, such as to estimate the refractive error from the AL or the AL from the refractive error and corneal power, which are more readily available. However, although these methods may work reasonably well on a population scale, Table 5 shows there may still be considerable errors for individual eyes due to the variations in ocular biometry and refractive powers, as well as the large overlap between refractive groups (Figures 1, 2, 3). These errors can run up to 0.3–1.5 mm, corresponding to relatively large errors in axial power of about 1–4 D. This is a clear indication that such methods will always be susceptible to a relatively large amount of uncertainty^68^ and are unlikely to be a suitable alternative to high‐quality measurements of SE, AL, P_c_, P_lb_ and ACD_tot_ (or lens power at the corneal plane instead of the latter two measures). However, it should be noted that if estimates are available for four out of these five parameters, then an exact estimate of the missing value can be calculated.^69^

**TABLE 5:** Overview of studies estimating axial length or refractive error from partial biometry.

First author	Year	Estimated parameter	Available parameters	*N*	Limits of agreement	Mean absolute error	*r* ^2^
Kim^70^	2019	AL	SE, r_ca_	696	[−0.74, 1.10] mm	–	0.935
Morgan^71^	2020	AL	SE, r_ca_	1046	±0.73 mm	–	0.83
		AL	SE	1046	±1.26 mm	–	0.57
Jeong^72^	2020	AL	Fundus image	1296	–	0.90 mm	0.67
Dong^73^	2021	AL	Fundus image	2811	–	0.56 mm	0.59
Queirós^74^	2022	AL	SE, r_ca_	1783	[−1.20, 0.70] mm	–	0.77
			Age, SE, P_c_	1783	–	–	0.798
Galvis^74^	2022	AL	SE, r_ca_	2129	[−1.56, 0.53] mm	–	–
Oh^75^	2023	AL	Fundus image	8254	–	0.74 mm	0.815
Lingham^54^	2024	AL	Age, sex, SE, P_c_	8135	–	0.31 mm	–
Noya‐Padin^76^	2024	AL	SE, P_c_, height	170	–	–	0.824

Abbreviations: AL, axial length; r_ca_, anterior corneal radius of curvature; P_c_, total corneal keratometry; SE, spherical equivalent.

Similar comments can be made about recent efforts to estimate the cycloplegic refractive error from non‐cycloplegic refractive error and biometry.^74^, ^77^, ^78^, ^79^, ^80^ While there is certainly a marked clinical advantage in not having to perform cycloplegia, both in terms of time management and patient comfort, the mean absolute error is typically of the order of 0.5–1.0 D (with much larger outliers in children), which is too large for reliable use in the follow‐up of myopia development.

### Correlations and ocular shape factors

As briefly hypothesised by Steiger,^7^ and later confirmed by many others,^9^, ^13^, ^15^, ^17^ strong correlations exist between the different ocular dimensions that, together, lead to the emergence of emmetropia.^13^, ^18^ Most of these relationships seem to change only moderately when considered for the entire population (Table 3), but the correlation values improve again when considered for each refractive group (Table S4).^13^ Furthermore, the slopes of the regression lines between the ocular dimensions show little variation (Figure 5), confirming earlier reports by Berg,^13^ van Alphen^81^ and Grossvenor.^82^ These observations all point to the fact that the overall layout of the eye follows a certain ocular shape factor,^81^, ^83^ a set of proportions for a human eye that is scalable to some degree. Hence, shorter emmetropic eyes will generally have smaller corneal and lenticular radii of curvature (i.e., more refractive power), while the opposite is found in longer emmetropic eyes (Figures 4 and 7). Given that the ocular parameters are all part of the same physical structure, that eye growth occurs according to a genetically pre‐programmed template and the great overall similarities between human eyes, such a shape factor would be expected.

**FIGURE 8:**
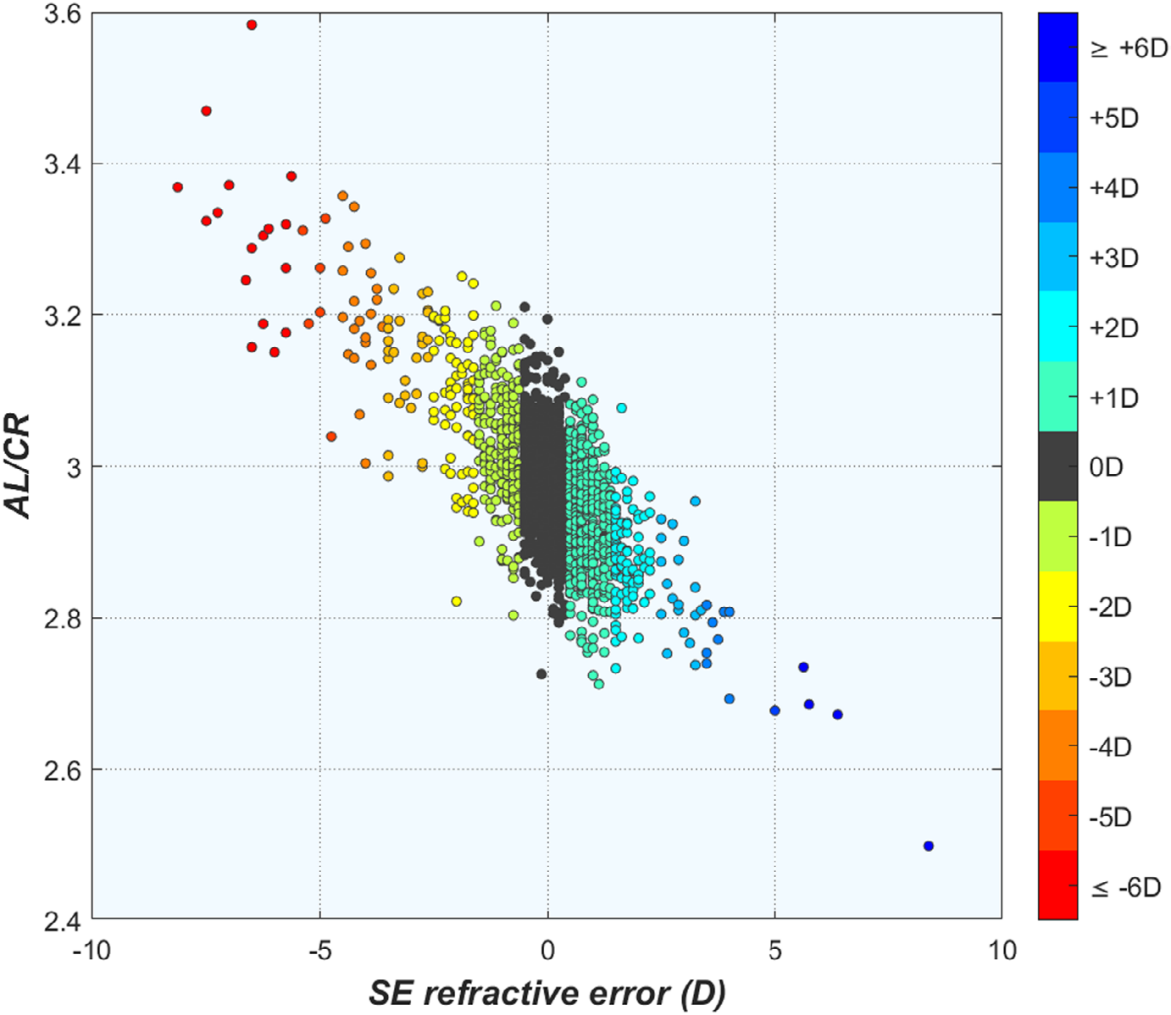
Axial length (AL) to corneal radius (CR) of curvature ratio as a function of refractive error. Emmetropic eyes are indicated by the dark grey markers. SE, spherical equivalent.

The first sign of a shape factor was found in 1886, when Bourgois and Tscherning^84^ were the first to notice that the mean corneal radius of curvature r_ca_ in their population was about three times shorter than that of the atypical AL. The value of this ratio, now commonly referred to as the AL/CR, is associated with the fact that about two‐thirds of the refractive power in the eye is provided by the cornea. This value was later confirmed by Stenström,^9^ Sorsby et al.^17^ and Grossvenor,^85^ who all saw it as evidence of a harmonious proportionality in the eye. More recently, the AL/CR ratio became an often‐used measure that correlates well with refractive error, where an AL/CR > 3 is typical for myopia. In practice, however, emmetropia can be found within an AL/CR range between 2.7 and 3.2, and again large overlaps are seen between refractive groups (Figure 8). This lack of specificity for emmetropia may be attributed to the large biometric variability,^86^ as well as the fact that the AL/CR does not consider the dioptric contributions of the crystalline lens, which is particularly important in childhood.^2^, ^3^, ^87^ Consequently, the clinical usefulness of the AL/CR ratio seems to be limited when compared with the ‘holistic’ approaches mentioned earlier.

Despite the existence of general ocular shape factors, there are also a few weaker correlations that make less sense at first sight. One such correlation is the decrease in corneal power with AL in individual eyes (Figure 5a), while the average value within each refractive subgroup appears to be increasing significantly with AL or myopia (Figure 2c).^88^ Since higher corneal powers are the exact opposite of what longer or myopic eyes need, Sorsby et al.^21^ deemed this observation paradoxical. However, as will be discussed later, this observation could also point to a more fundamental aspect of refractive development leading to myopia.

**FIGURE 9:**
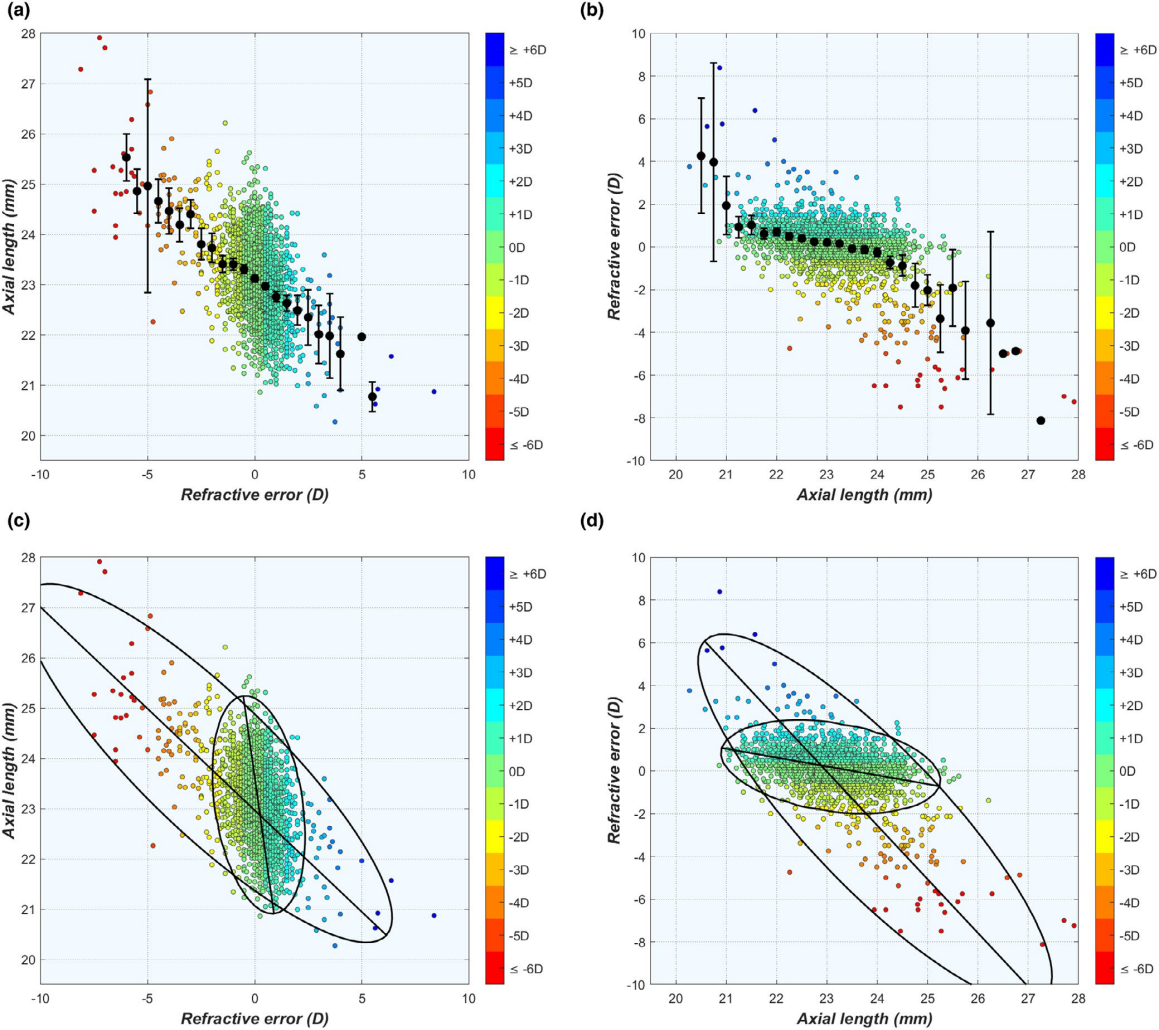
(a) Axial length (AL) plotted as a function of the spherical equivalent (SE) refractive error in bins of 0.5 D. (b) SE plotted as function of AL in bins of 0.25 mm. (c, d) As for (a, b), fitted with bigaussian ellipses representing the Regulated and Dysregulated subgroups.

Shape factors can also help to understand Sorsby's concepts of correlation ametropia and component ametropia, where the biometric parameters are relatively close to normal emmetropic values but match one another poorly and where an element (typically the AL) lies outside the emmetropic range. The large overlaps between biometric values across refractive groups within a range of ±3 D (Figure 2) seem to confirm this proposal, and the breakdown of the linear relationships between the ocular dimension for ALs beyond 27 mm (Figure 3) does indeed seem to confirm Sorsby's ideas. However, Carroll^22^ demonstrated that the term correlation ametropia makes no sense from a statistical point of view as, for example, plots of AL as a function of binned refractive error show a linear relationship,^22^ while the plot of the inverse more closely resembles a beach chair with a broad plateau for ALs between 21 and 25 mm, where most eyes are emmetropic (Figure 9a,b). As the range of the plateau roughly corresponds with the emmetropic AL range, this may suggest that most eyes in that range follow a specific (near) emmetropic shape factor, while the eyes outside that range follow a different shape factor.

Looking at the relationship between AL and refractive error in terms of the bigaussian analysis, new and valuable information can also be found. The plateau in Figure 9b is dominated by the Regulated subgroup, while outside that range the Dysregulated subgroup is dominant (Figure 9c,d). Following Sorsby's definition, this suggests that the Regulated group corresponds to those eyes with emmetropia and correlation ametropia that follow a specific shape factor, while the Dysregulated eyes are those eyes with component ametropia that deviate from that particular shape factor. This corresponds to the definition of both groups in the section entitled ‘Bigaussian analysis’ within the results section, which allows assigning individual eyes to either subgroup within a certain population, while it also explains the existence of the ‘lucky’ Dysregulated eyes that have a (near) emmetropic refractive error despite one or more deviated biometric values (Figure 6e). Consequently, distinguishing between Regulated and Dysregulated eyes may prove a better classification system than axial or refractive myopia, as all refractive states have mixed origins.

As the Regulated group consists of considerably more than the emmetropic eyes, the question remains why this group is so broad [−1.46 D to +2.20 D]. Speculatively, this may be associated with the accuracy of the emmetropisation process across the general population or perhaps be aligned with variations in the individual tolerances to retinal blur. In that view, the Dysregulated eyes are then outliers from the normal, Regulated eyes, considered across all biometric parameters.

### Scaling eyes

One might expect that the ocular dimensions of emmetropic eyes scale proportionally according to their AL. However, this is not the case, as the lens is thinner in longer eyes to provide the lower optical power required (Figure 7). Here the position of the anterior lens surface shifts towards the retina relatively slowly in longer eyes, while the posterior surface moves quickly towards the cornea, suggesting that the posterior lens surface may flatten more for longer ALs than the anterior surface.

In terms of power, the refractive contributions of the cornea and lens are 69.9% versus 30.1%, respectively, for a 21‐mm emmetropic eye, while for a 26‐mm eye the respective values are 63.4% versus 26.6%. This shows how longer and shorter eyes have a slightly different balance between the refractive elements, which could have repercussions for myopia studies. Meanwhile, for intraocular lens power calculations, these changes in lens position with AL will affect the location of the crystalline lens equator that likely determines the effective postoperative lens position inside the eye. This could explain why long or short eyes do not always have the best possible postoperative outcomes following cataract surgery.^89^, ^90^

### Refractive development

It has been established^2^, ^3^, ^32^ that refractive development entails a complex choreography by the ocular components to reach and maintain emmetropia during continued eye growth. As fairly large biometric variations are already seen at birth,^91^, ^92^ it is clear that the details of this process must vary somewhat between individual eyes for it to be successful. How this occurs exactly, either at a population or individual level, remains unclear.

Axial eye growth is affected by genetic and hereditary influences^93^ that predetermine mechanical factors such as scleral stiffness^94^ or intraocular pressure,^95^, ^96^ body size and perhaps the sensitivity of the retinal response to certain visual inputs that could predispose an eye to myopic growth. This genetic basis combines with growth changes due to the child's visual behaviour (e.g., spending much time indoors or doing excessive near work)^97^ and visual environment (e.g., urban or indoor areas with low ambient light^98^ and relatively few spatial frequencies^99^). Meanwhile, as the eye grows, the cornea and lens must lose power to maintain emmetropia by compensating for the loss in axial power P_ax_. The corneal power will stabilise into a (near) constant value by the age of 3 years,^3^, ^100^, ^101^, ^102^ so most of this compensation must come from the crystalline lens,^2^, ^55^, ^103^ a fact first theorised by Straub^6^ and later confirmed by Sorsby et al.^19^ Consequently, the ability of an eye to emmetropise is largely determined by the ability of the crystalline lens to lose power as the eye grows.

There are two ways in which the lens could reduce its power, either through internal remodelling or mechanical stretching. This internal remodelling includes the growth and compaction of lens epithelial cells that will develop into specialised lens fibres, a process that begins in utero and continues throughout life. This causes the lens to gradually thicken with time, but also the formation of a gradient refractive index.^103^ Meanwhile, given that the lens is suspended within a ring‐shaped ciliary muscle that is in turn attached to the sclera, the lens is being stretched radially in all directions when the eye globe expands. This could cause the lens diameter to increase (during the first years after birth), while its surfaces become flatter and its thickness decreases, typically between birth and up to the age of 10–12 years.^103^ These two influences have opposite effects on the lens power. The thicker, rounder lens shape is associated with higher power, while stretching flattens the lens, thus lowering its refractive power.^2^ The increase in stretching ends when the eyeball stops expanding, leaving continued lens development entirely in the hands of its internal processes that will continue to not only increase the thickness and surface curvatures but also alter the gradient index profile to become steeper. Altogether, this leads to a smooth loss of lens power over time that follows the eye growth.^3^

In cases of accelerated axial growth, the lens power loss will also accelerate to preserve emmetropia as much as possible, but at some point it will reach a state in which it is no longer able to lose power efficiently (or not at all).^104^, ^105^ If and when this occurs, the eye will become myopic. This may be the reason why the average corneal power is higher in myopic than in emmetropic eyes (Figure 2), as higher corneal powers may create a greater risk for myopia as it requires the crystalline lens to lose more power for the same AL than flatter corneas. This could also partially explain why girls, who have shorter eyes and steeper corneas, become more myopic^55^, ^105^ at a faster rate^106^, ^107^ than boys during childhood. However, the onset of puberty and growth spurts may also play a role in these sex differences,^108^ and after the age of 14 years, no significant sex‐based differences remain.^55^ Meanwhile, deeper anterior chambers can reduce the effective power of the lens and have recently been identified as a potential risk factor for faster axial growth.^109^

Despite all these observations and theories, many important details about refractive development remain unknown. Consequently, current methods to predict future eye growth or development of refractive errors remain imprecise, pointing to a lack of suitable parameters to discriminate those at risk of myopia development from the individuals who will remain emmetropic.

### Limitations

The limitations of the current analysis mainly lie in the assumptions made in the bigaussian analysis. One such assumption was that the Regulated and Dysregulated groups could be delineated within 2 D ellipses based on pairs of parameters, rather than a single 5 D ellipsoid, which was found to be too complicated to implement due to the complicated parameter transformations required. This may perhaps have misclassified several individual eyes, but was unlikely to affect the overall results of the analysis. A second assumption was that the bigaussian analysis can be done adequately over five parameters, instead of using eight parameters (including P_ca_, P_cp_ and CCT) as the latter fit had trouble converging despite attempting many combinations of boundary conditions. Regardless, the results of the eight‐parameter fit would likely not differ much from that presented above. Furthermore, while the results presented here match many of the prior reports in the literature, another possible limitation is that the specific sample used here may not represent eyes of other ethnicities and other demographic characteristics, including age.

## CONCLUSIONS

By analysing the biometric and refraction data of 2000 healthy adult eyes, this work confirms prior observations of a large overlap in ocular biometry between refractive groups. This is a clear indication that characterising the refractive condition of an eye based on incomplete biometric values (e.g., the combination of refractive error and AL) provides insufficient information to follow up either emmetropisation or myopia development adequately. Rather, the refractive contributions of the cornea and crystalline lens must also be considered.

This work also introduces a new method to distinguish individual Regulated and Dysregulated eyes based on their full ocular biometry in a way that matches with the refractive error distribution. When applied to longitudinal data, this method could be a useful tool for a detailed biometric analysis of how the different refractive groups emerge over time.

## AUTHOR CONTRIBUTIONS

**Jos J. Rozema:** Conceptualization (lead); data curation (equal); formal analysis (lead); investigation (lead); methodology (lead); software (lead); visualization (lead); writing – original draft (lead); writing – review and editing (lead). **Mohammad Hassan Emamian:** Data curation (equal); funding acquisition (equal); project administration (equal); resources (equal); writing – review and editing (supporting). **Hassan Hashemi:** Data curation (equal); funding acquisition (equal); project administration (equal); resources (equal); writing – review and editing (supporting). **Akbar Fotouhi:** Data curation (equal); funding acquisition (equal); project administration (equal); resources (equal); writing – review and editing (supporting).

## FUNDING INFORMATION

This project was funded in part by the Noor Ophthalmology Research Centre and the Shahroud University of Medical Sciences (project number: 960351).

## CONFLICT OF INTEREST STATEMENT

None.

## Supporting information


Appendix S1.
