# Supplementary material for: Refractive development III: Variations in emmetropia and ametropia
Source: Ophthalmic Physiol Opt. 2025 May 19;45(4):1004–24. doi: 10.1111/opo.13516 (PMC12087875; doi:10.1111/opo.13516)
Supplement: Supplementary file 1 — Appendix S1. [file OPO-45-1004-s001.pptx]

## Slide 1
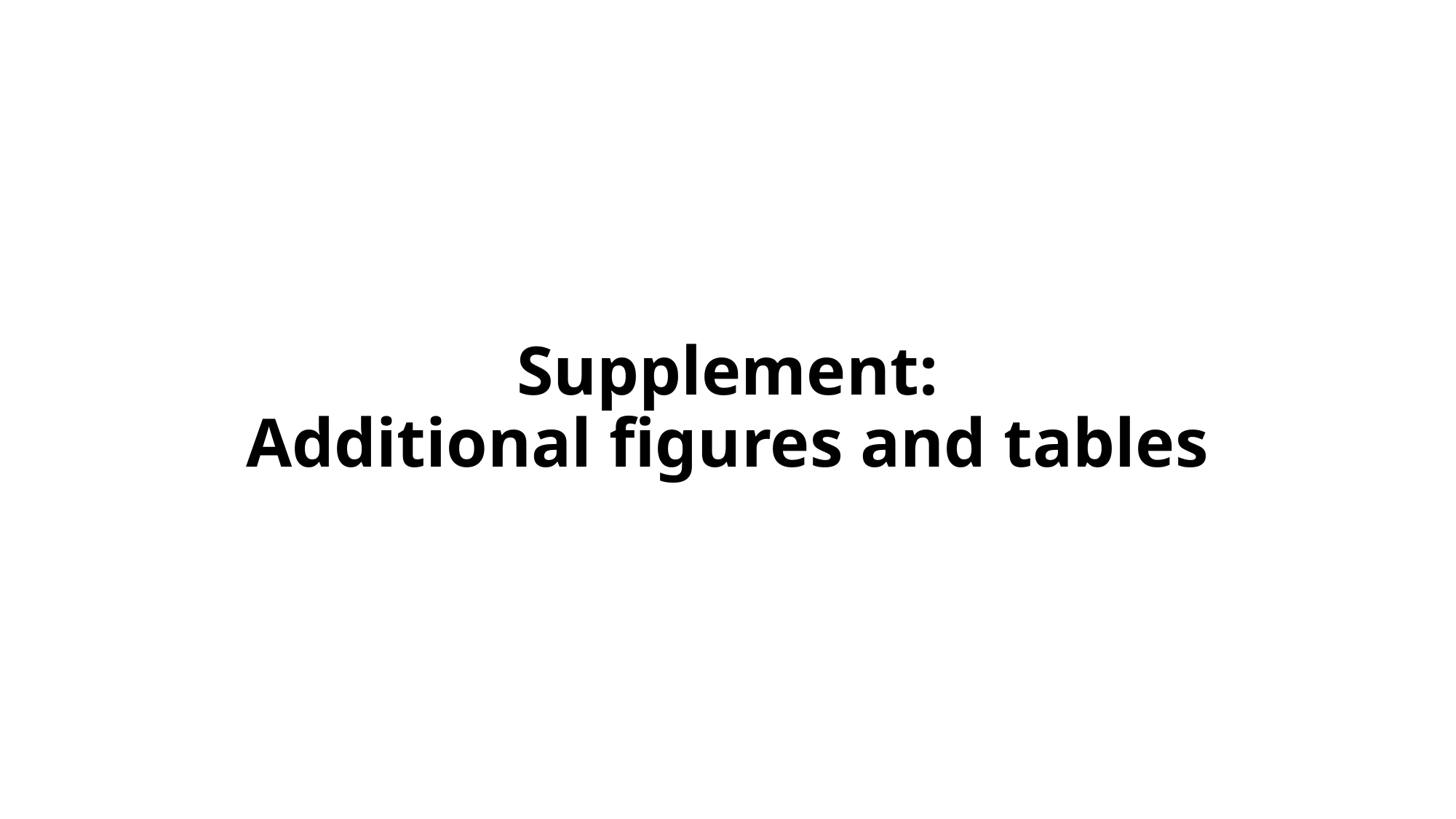

# Supplement: Additional figures and tables

## Slide 2
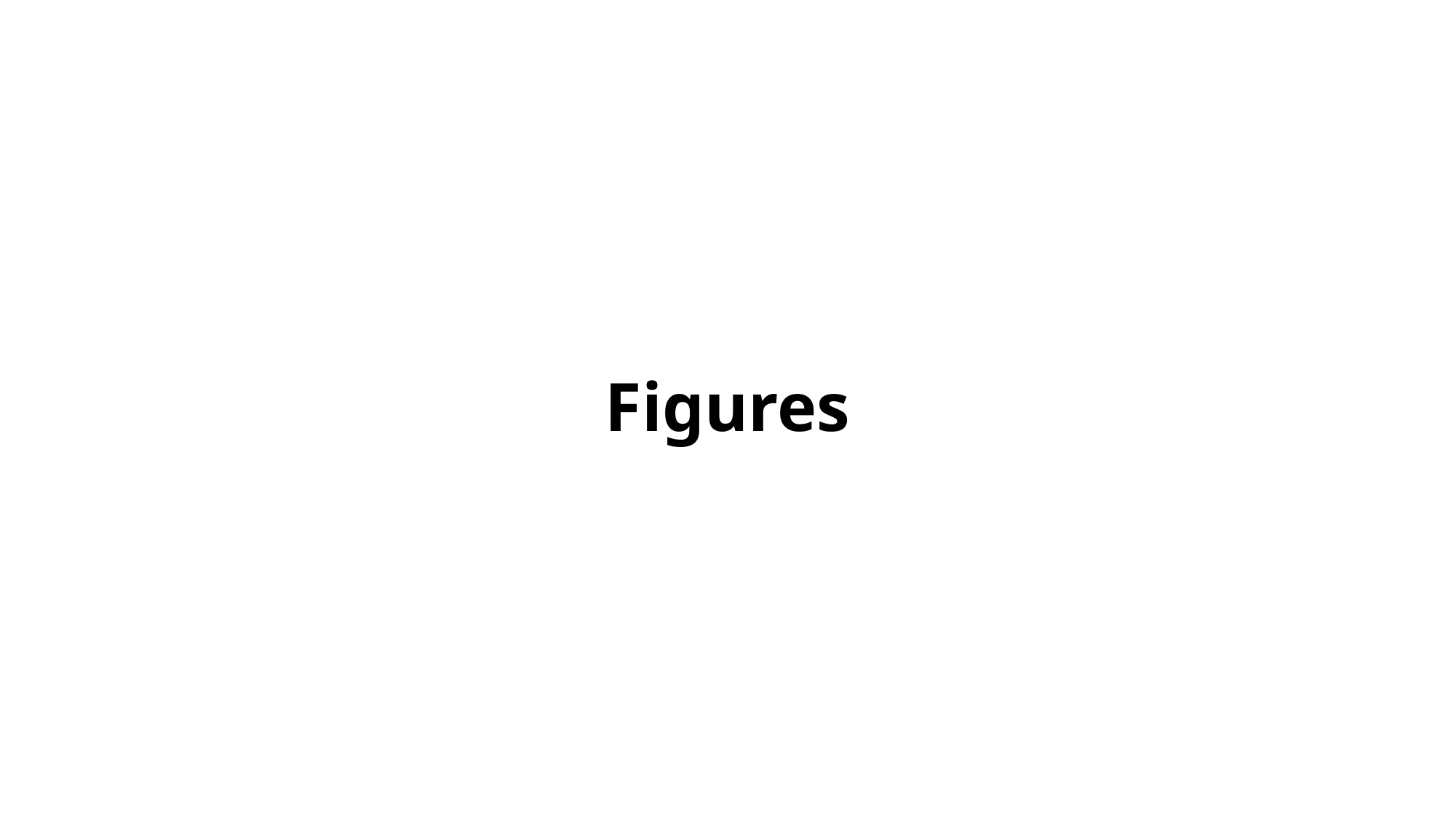

# Figures

## Slide 3
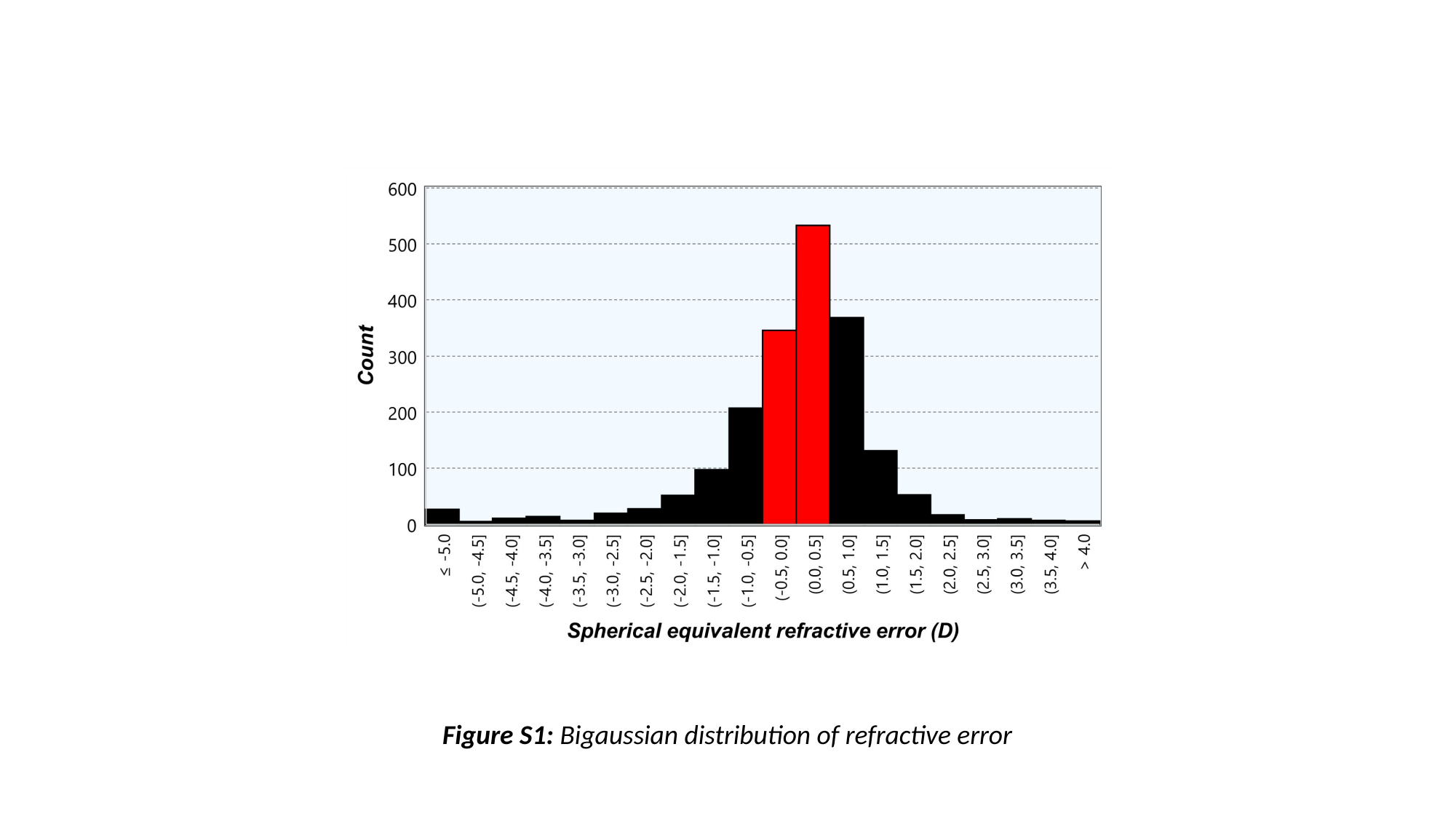

Figure S1: Bigaussian distribution of refractive error

## Slide 4
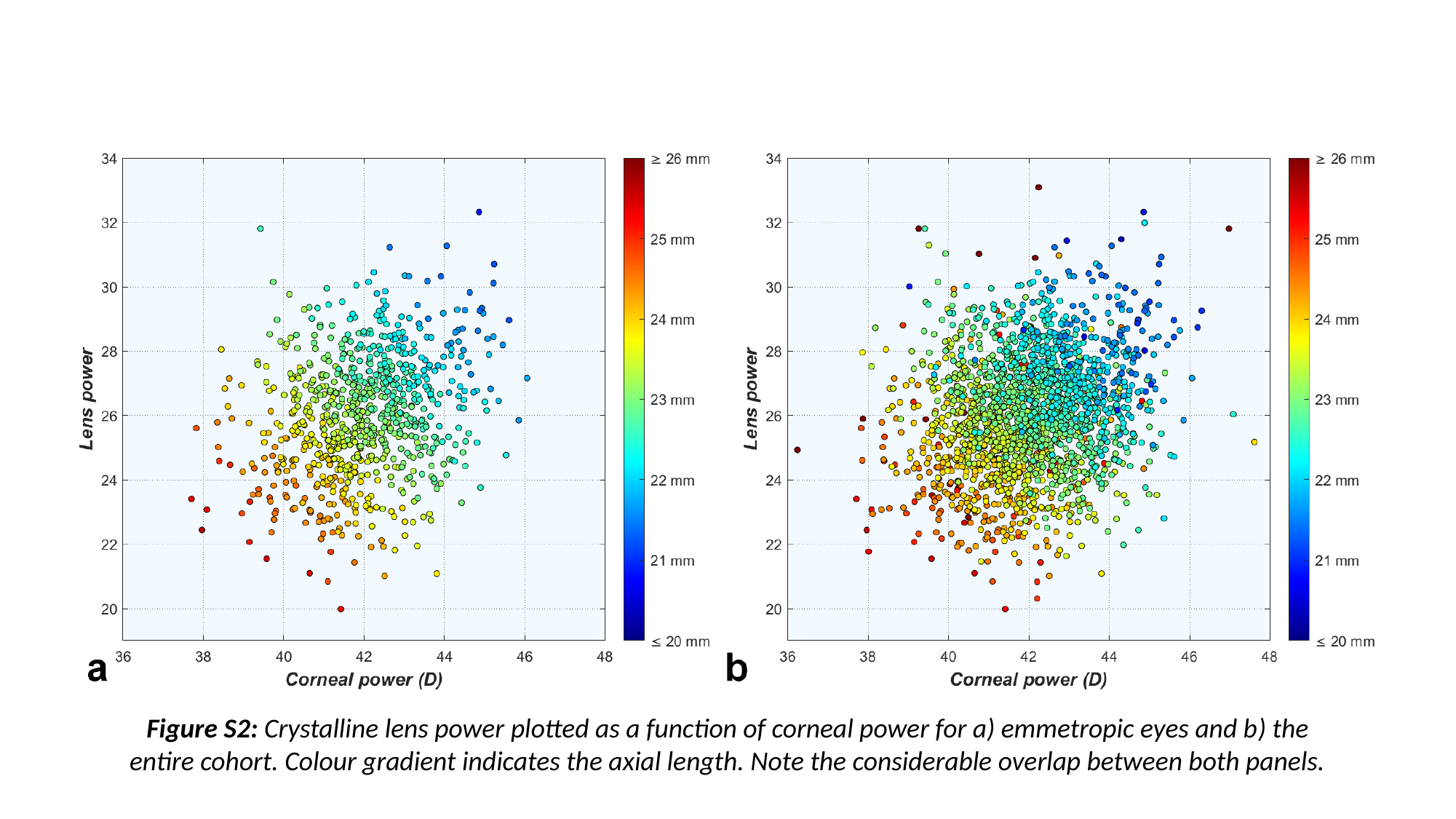

Figure S2: Crystalline lens power plotted as a function of corneal power for a) emmetropic eyes and b) the entire cohort. Colour gradient indicates the axial length. Note the considerable overlap between both panels.

## Slide 5
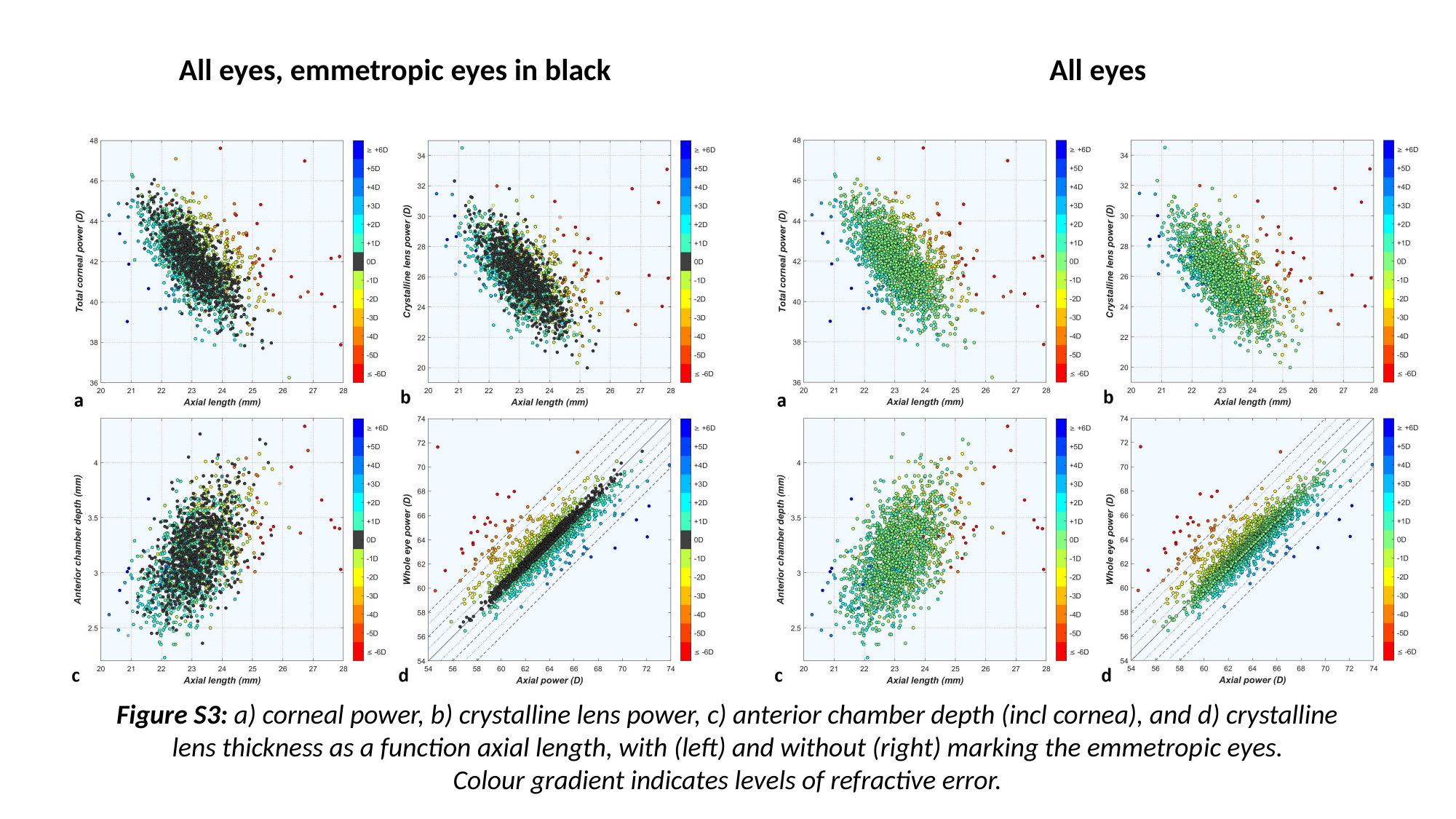

All eyes, emmetropic eyes in black
All eyes
Figure S3: a) corneal power, b) crystalline lens power, c) anterior chamber depth (incl cornea), and d) crystalline lens thickness as a function axial length, with (left) and without (right) marking the emmetropic eyes.
Colour gradient indicates levels of refractive error.

## Slide 6
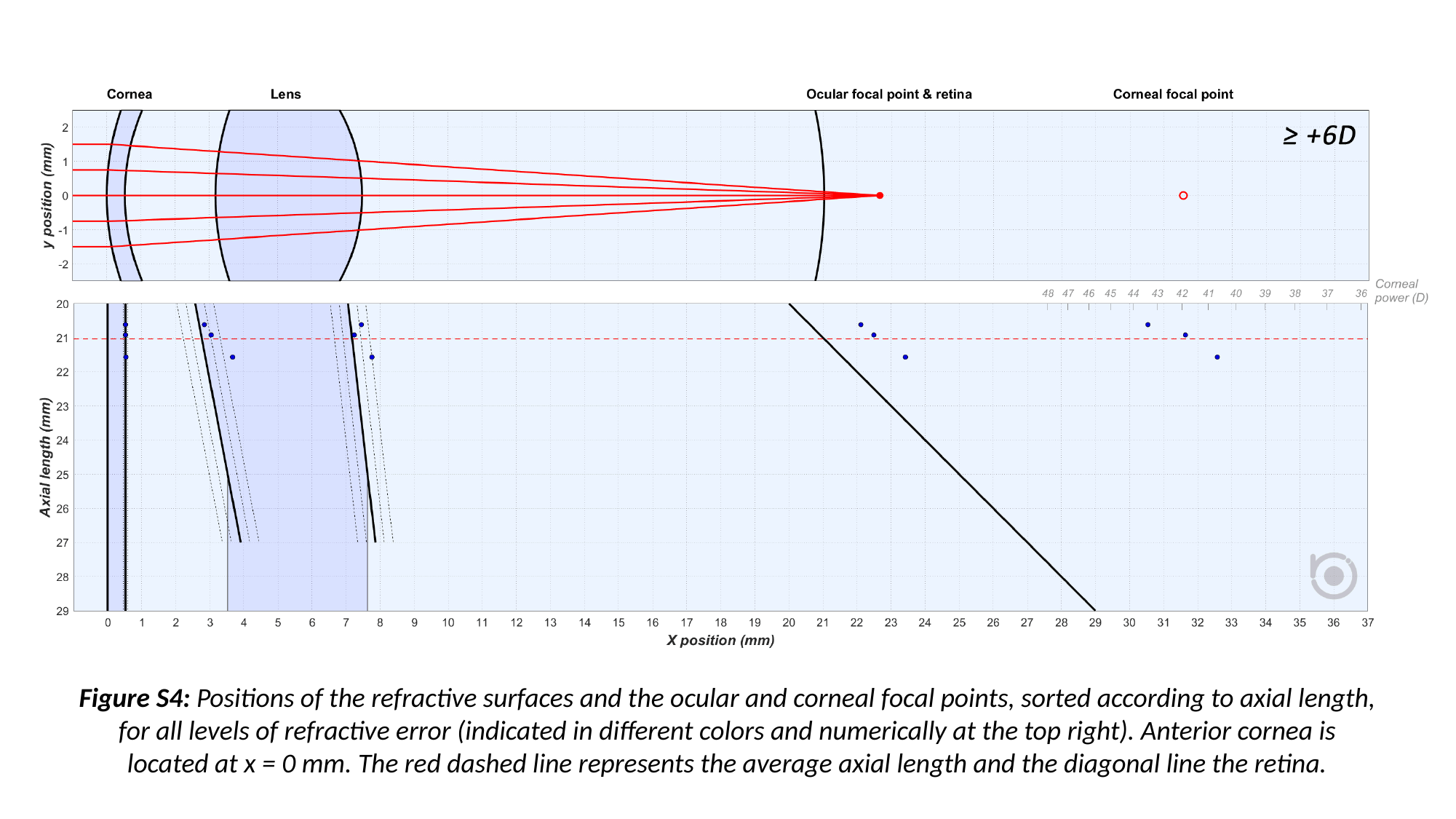

Figure S4: Positions of the refractive surfaces and the ocular and corneal focal points, sorted according to axial length, for all levels of refractive error (indicated in different colors and numerically at the top right). Anterior cornea is located at x = 0 mm. The red dashed line represents the average axial length and the diagonal line the retina.

## Slide 7
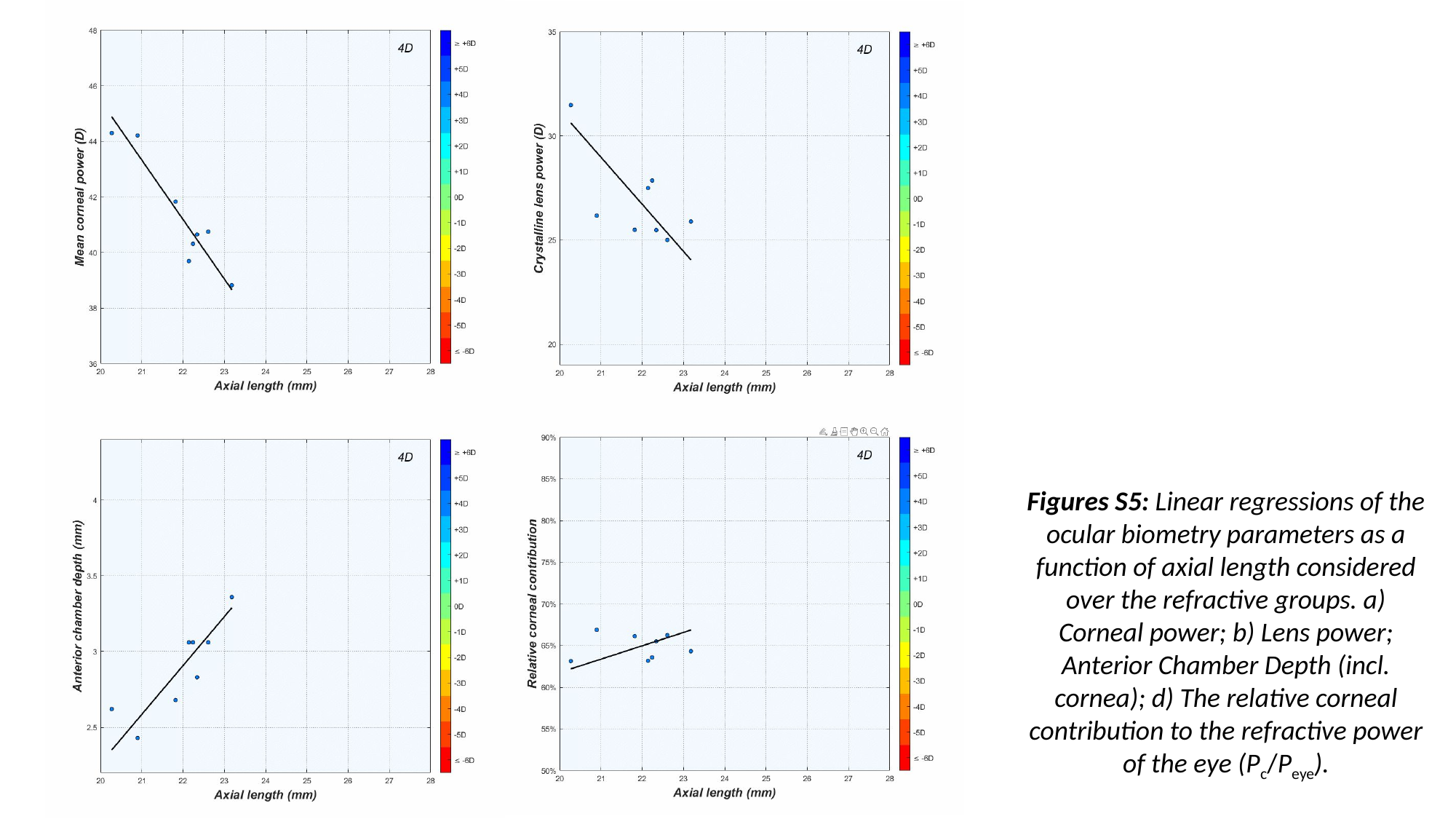

Figures S5: Linear regressions of the ocular biometry parameters as a function of axial length considered over the refractive groups. a) Corneal power; b) Lens power; Anterior Chamber Depth (incl. cornea); d) The relative corneal contribution to the refractive power of the eye (Pc/Peye).

## Slide 8
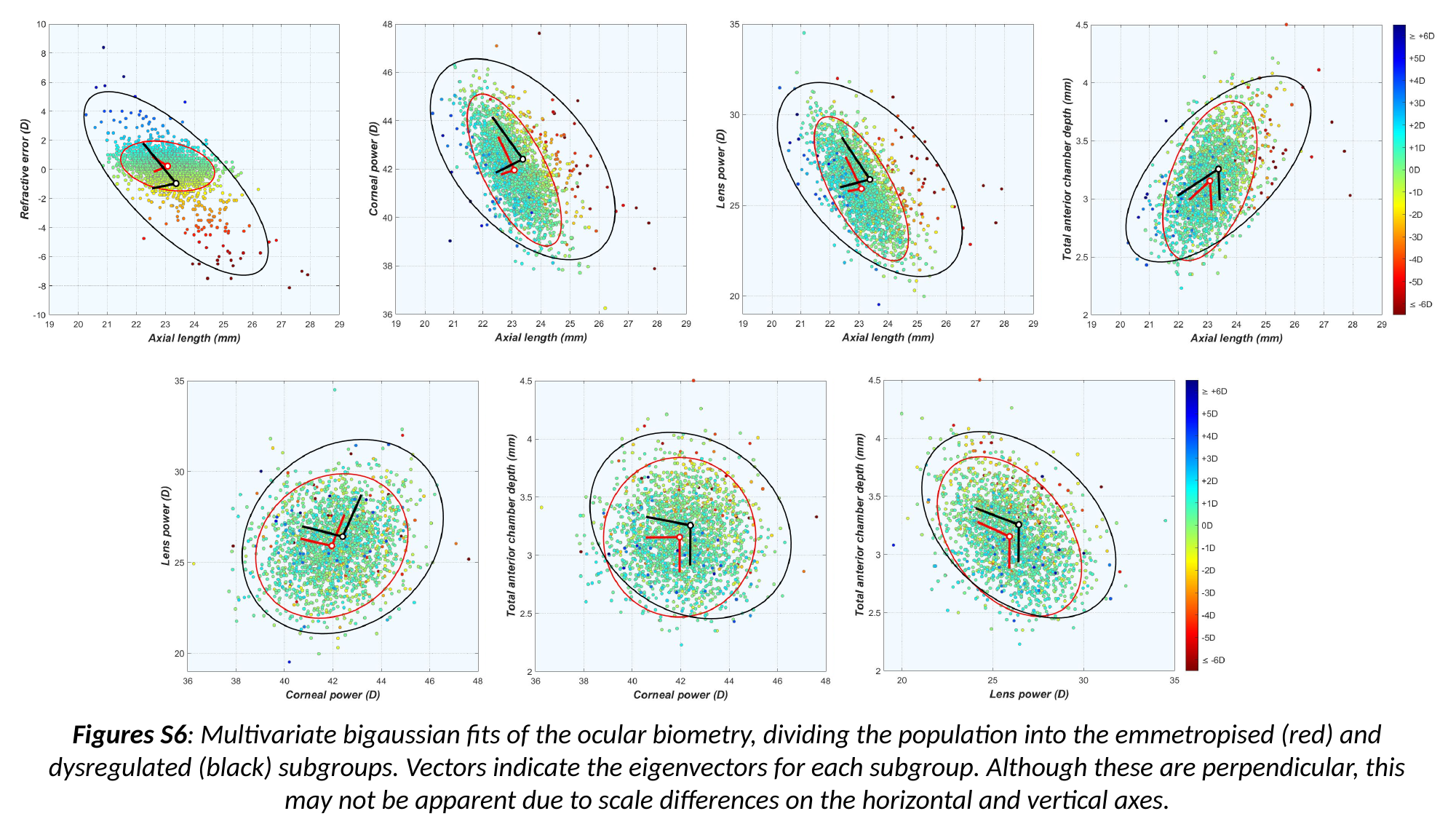

Figures S6: Multivariate bigaussian fits of the ocular biometry, dividing the population into the emmetropised (red) and dysregulated (black) subgroups. Vectors indicate the eigenvectors for each subgroup. Although these are perpendicular, this may not be apparent due to scale differences on the horizontal and vertical axes.

## Slide 9
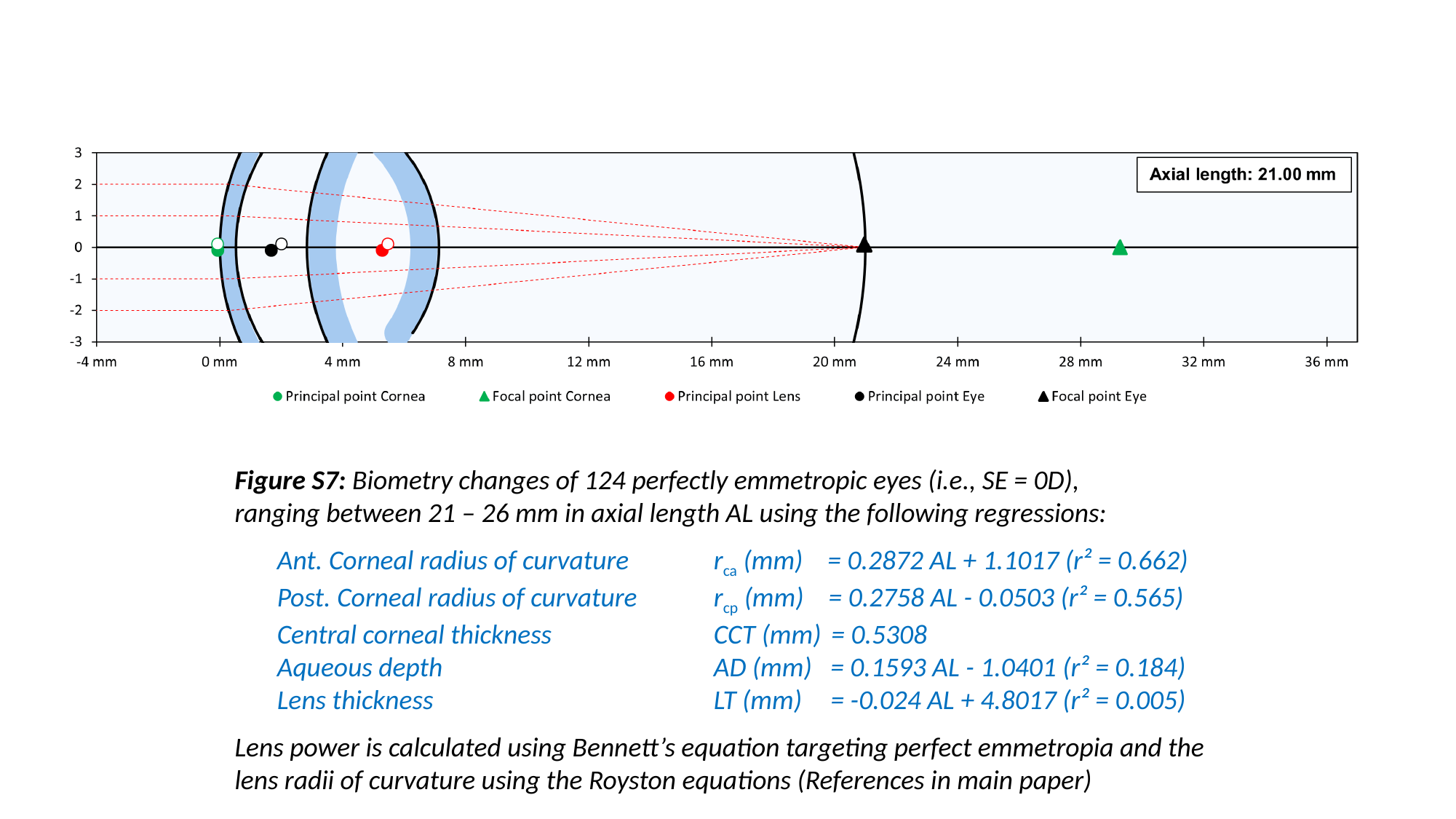

Figure S7: Biometry changes of 124 perfectly emmetropic eyes (i.e., SE = 0D),
ranging between 21 – 26 mm in axial length AL using the following regressions:
Ant. Corneal radius of curvature	rca (mm) = 0.2872 AL + 1.1017 (r² = 0.662)
Post. Corneal radius of curvature	rcp (mm) = 0.2758 AL - 0.0503 (r² = 0.565)
Central corneal thickness		CCT (mm) = 0.5308
Aqueous depth 			AD (mm) = 0.1593 AL - 1.0401 (r² = 0.184)
Lens thickness			LT (mm) = -0.024 AL + 4.8017 (r² = 0.005)
Lens power is calculated using Bennett’s equation targeting perfect emmetropia and the lens radii of curvature using the Royston equations (References in main paper)

## Slide 10
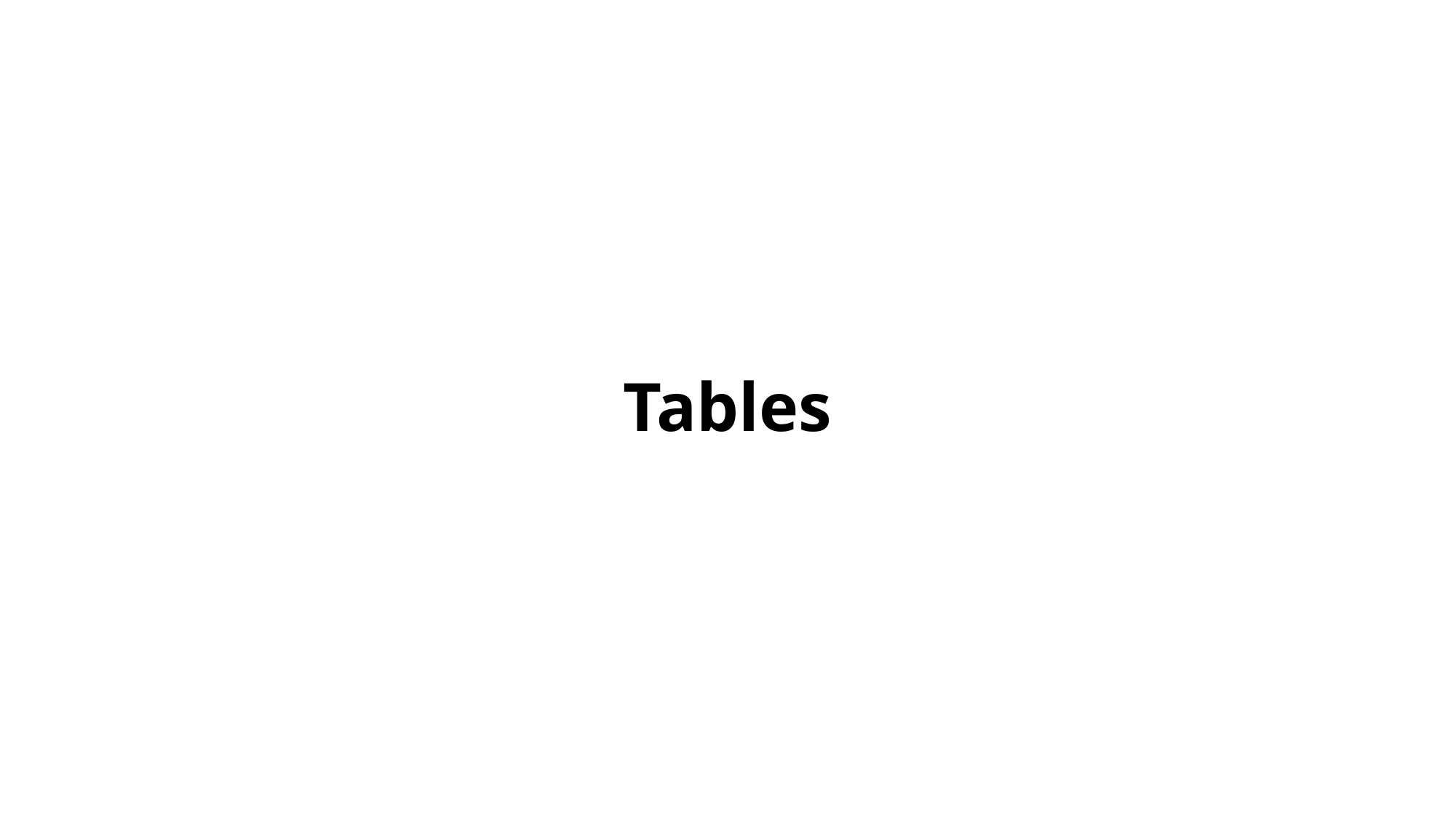

# Tables

## Slide 11
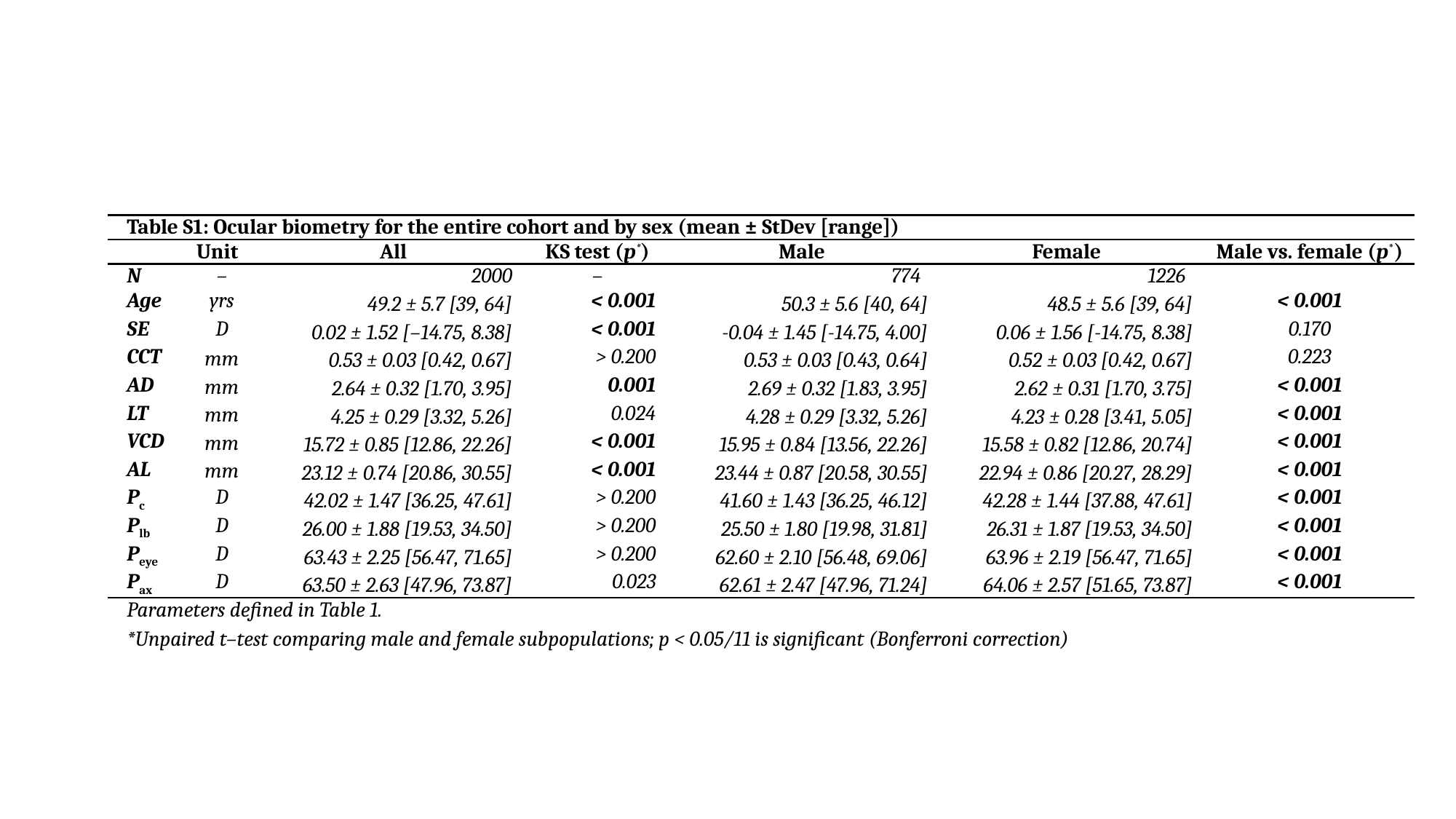

| | | | | | | |
| --- | --- | --- | --- | --- | --- | --- |
| Table S1: Ocular biometry for the entire cohort and by sex (mean ± StDev [range]) | | | | | | |
| | Unit | All | KS test (p\*) | Male | Female | Male vs. female (p\*) |
| N | – | 2000 | – | 774 | 1226 | |
| Age | yrs | 49.2 ± 5.7 [39, 64] | < 0.001 | 50.3 ± 5.6 [40, 64] | 48.5 ± 5.6 [39, 64] | < 0.001 |
| SE | D | 0.02 ± 1.52 [–14.75, 8.38] | < 0.001 | -0.04 ± 1.45 [-14.75, 4.00] | 0.06 ± 1.56 [-14.75, 8.38] | 0.170 |
| CCT | mm | 0.53 ± 0.03 [0.42, 0.67] | > 0.200 | 0.53 ± 0.03 [0.43, 0.64] | 0.52 ± 0.03 [0.42, 0.67] | 0.223 |
| AD | mm | 2.64 ± 0.32 [1.70, 3.95] | 0.001 | 2.69 ± 0.32 [1.83, 3.95] | 2.62 ± 0.31 [1.70, 3.75] | < 0.001 |
| LT | mm | 4.25 ± 0.29 [3.32, 5.26] | 0.024 | 4.28 ± 0.29 [3.32, 5.26] | 4.23 ± 0.28 [3.41, 5.05] | < 0.001 |
| VCD | mm | 15.72 ± 0.85 [12.86, 22.26] | < 0.001 | 15.95 ± 0.84 [13.56, 22.26] | 15.58 ± 0.82 [12.86, 20.74] | < 0.001 |
| AL | mm | 23.12 ± 0.74 [20.86, 30.55] | < 0.001 | 23.44 ± 0.87 [20.58, 30.55] | 22.94 ± 0.86 [20.27, 28.29] | < 0.001 |
| Pc | D | 42.02 ± 1.47 [36.25, 47.61] | > 0.200 | 41.60 ± 1.43 [36.25, 46.12] | 42.28 ± 1.44 [37.88, 47.61] | < 0.001 |
| Plb | D | 26.00 ± 1.88 [19.53, 34.50] | > 0.200 | 25.50 ± 1.80 [19.98, 31.81] | 26.31 ± 1.87 [19.53, 34.50] | < 0.001 |
| Peye | D | 63.43 ± 2.25 [56.47, 71.65] | > 0.200 | 62.60 ± 2.10 [56.48, 69.06] | 63.96 ± 2.19 [56.47, 71.65] | < 0.001 |
| Pax | D | 63.50 ± 2.63 [47.96, 73.87] | 0.023 | 62.61 ± 2.47 [47.96, 71.24] | 64.06 ± 2.57 [51.65, 73.87] | < 0.001 |
| Parameters defined in Table 1. \*Unpaired t–test comparing male and female subpopulations; p < 0.05/11 is significant (Bonferroni correction) | | | | | | |

## Slide 12
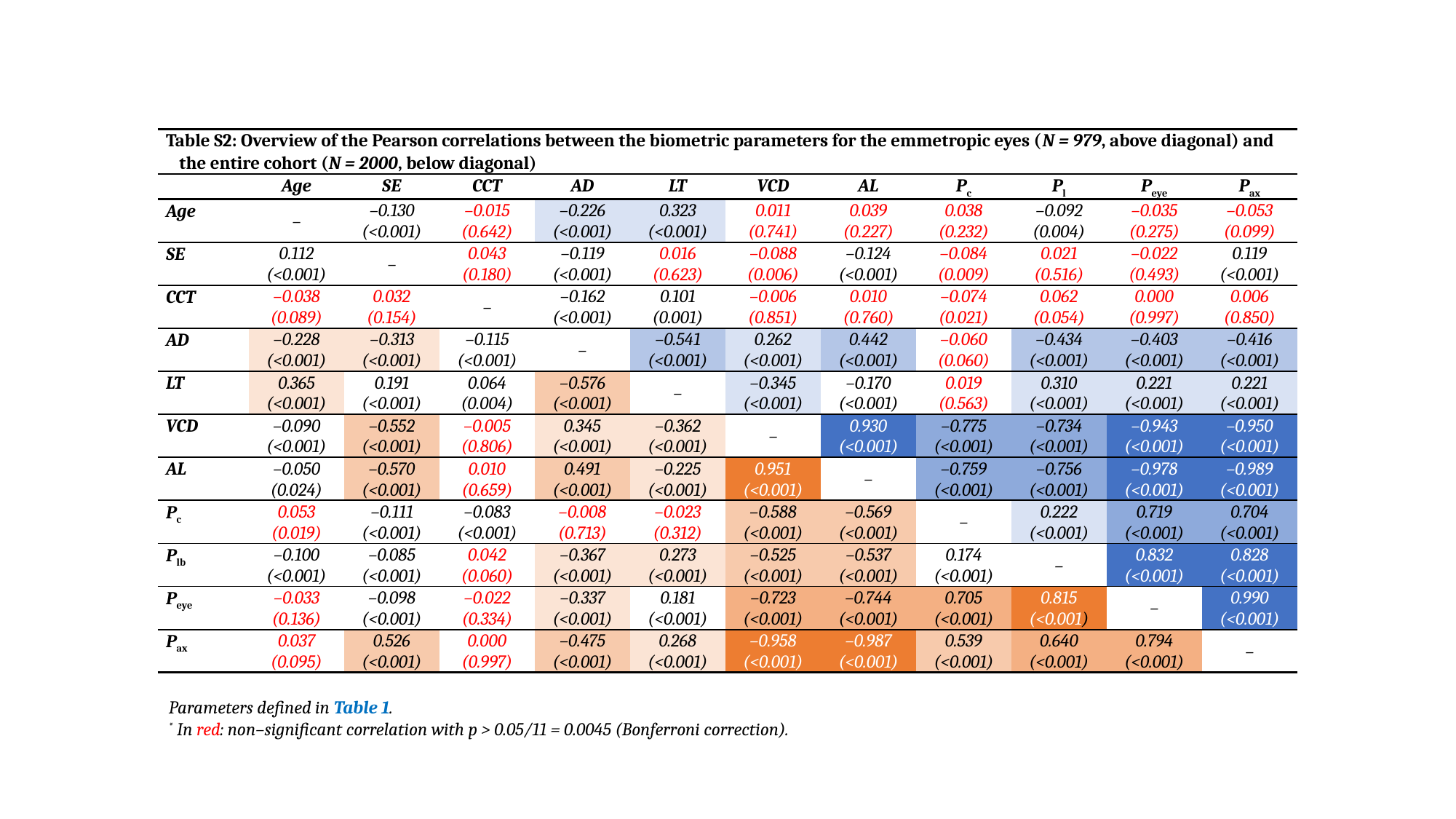

| Table S2: Overview of the Pearson correlations between the biometric parameters for the emmetropic eyes (N = 979, above diagonal) and the entire cohort (N = 2000, below diagonal) | | | | | | | | | | | |
| --- | --- | --- | --- | --- | --- | --- | --- | --- | --- | --- | --- |
| | Age | SE | CCT | AD | LT | VCD | AL | Pc | Pl | Peye | Pax |
| Age | – | –0.130 (<0.001) | –0.015 (0.642) | –0.226 (<0.001) | 0.323 (<0.001) | 0.011 (0.741) | 0.039 (0.227) | 0.038 (0.232) | –0.092 (0.004) | –0.035 (0.275) | –0.053 (0.099) |
| SE | 0.112 (<0.001) | – | 0.043 (0.180) | –0.119 (<0.001) | 0.016 (0.623) | –0.088 (0.006) | –0.124 (<0.001) | –0.084 (0.009) | 0.021 (0.516) | –0.022 (0.493) | 0.119 (<0.001) |
| CCT | –0.038 (0.089) | 0.032 (0.154) | – | –0.162 (<0.001) | 0.101 (0.001) | –0.006 (0.851) | 0.010 (0.760) | –0.074 (0.021) | 0.062 (0.054) | 0.000 (0.997) | 0.006 (0.850) |
| AD | –0.228 (<0.001) | –0.313 (<0.001) | –0.115 (<0.001) | – | –0.541 (<0.001) | 0.262 (<0.001) | 0.442 (<0.001) | –0.060 (0.060) | –0.434 (<0.001) | –0.403 (<0.001) | –0.416 (<0.001) |
| LT | 0.365 (<0.001) | 0.191 (<0.001) | 0.064 (0.004) | –0.576 (<0.001) | – | –0.345 (<0.001) | –0.170 (<0.001) | 0.019 (0.563) | 0.310 (<0.001) | 0.221 (<0.001) | 0.221 (<0.001) |
| VCD | –0.090 (<0.001) | –0.552 (<0.001) | –0.005 (0.806) | 0.345 (<0.001) | –0.362 (<0.001) | – | 0.930 (<0.001) | –0.775 (<0.001) | –0.734 (<0.001) | –0.943 (<0.001) | –0.950 (<0.001) |
| AL | –0.050 (0.024) | –0.570 (<0.001) | 0.010 (0.659) | 0.491 (<0.001) | –0.225 (<0.001) | 0.951 (<0.001) | – | –0.759 (<0.001) | –0.756 (<0.001) | –0.978 (<0.001) | –0.989 (<0.001) |
| Pc | 0.053 (0.019) | –0.111 (<0.001) | –0.083 (<0.001) | –0.008 (0.713) | –0.023 (0.312) | –0.588 (<0.001) | –0.569 (<0.001) | – | 0.222 (<0.001) | 0.719 (<0.001) | 0.704 (<0.001) |
| Plb | –0.100 (<0.001) | –0.085 (<0.001) | 0.042 (0.060) | –0.367 (<0.001) | 0.273 (<0.001) | –0.525 (<0.001) | –0.537 (<0.001) | 0.174 (<0.001) | – | 0.832 (<0.001) | 0.828 (<0.001) |
| Peye | –0.033 (0.136) | –0.098 (<0.001) | –0.022 (0.334) | –0.337 (<0.001) | 0.181 (<0.001) | –0.723 (<0.001) | –0.744 (<0.001) | 0.705 (<0.001) | 0.815 (<0.001) | – | 0.990 (<0.001) |
| Pax | 0.037 (0.095) | 0.526 (<0.001) | 0.000 (0.997) | –0.475 (<0.001) | 0.268 (<0.001) | –0.958 (<0.001) | –0.987 (<0.001) | 0.539 (<0.001) | 0.640 (<0.001) | 0.794 (<0.001) | – |
Parameters defined in Table 1.
* In red: non–significant correlation with p > 0.05/11 = 0.0045 (Bonferroni correction).

## Slide 13
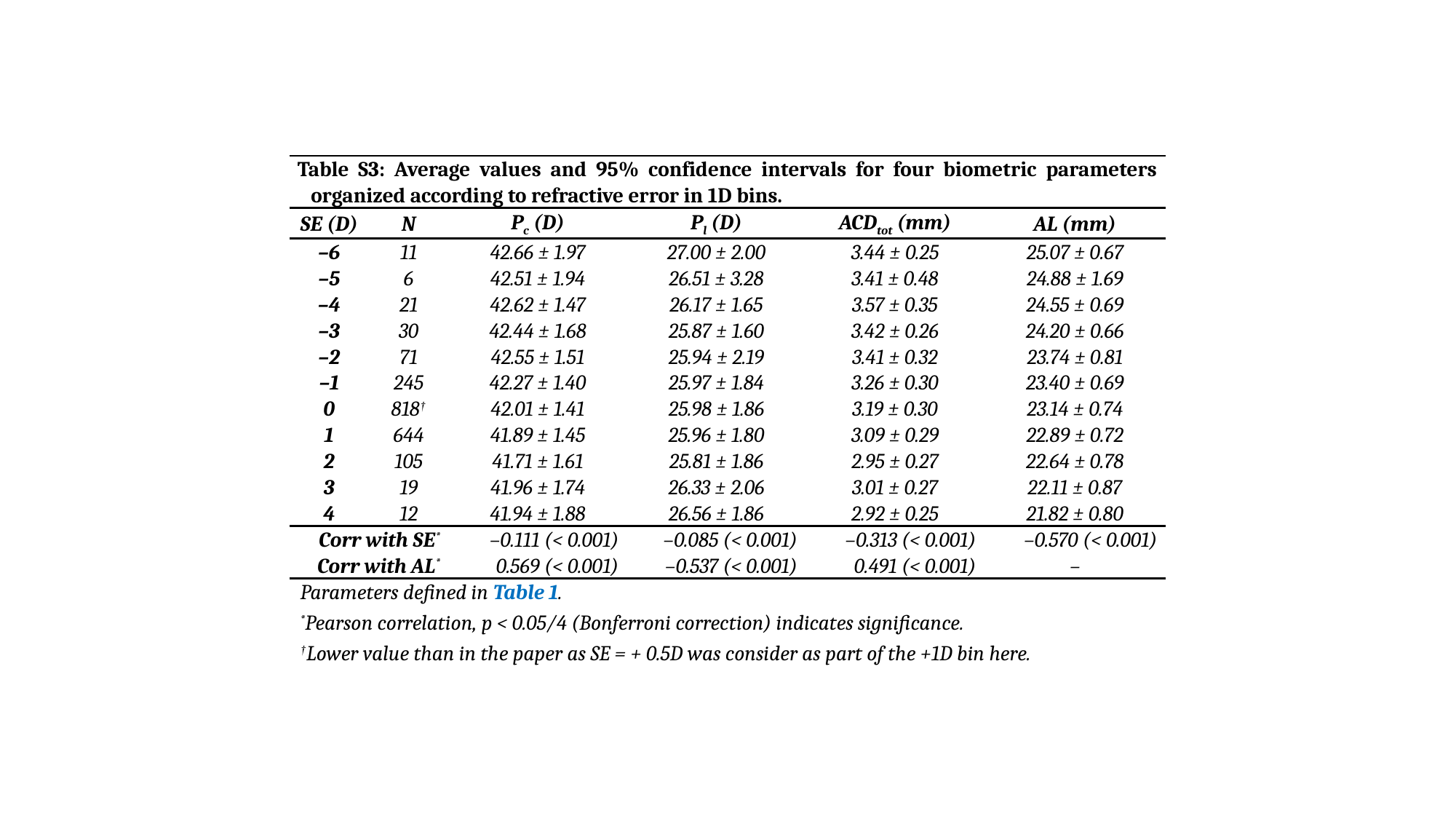

| Table S3: Average values and 95% confidence intervals for four biometric parameters organized according to refractive error in 1D bins. | | | | | |
| --- | --- | --- | --- | --- | --- |
| SE (D) | N | Pc (D) | Pl (D) | ACDtot (mm) | AL (mm) |
| –6 | 11 | 42.66 ± 1.97 | 27.00 ± 2.00 | 3.44 ± 0.25 | 25.07 ± 0.67 |
| –5 | 6 | 42.51 ± 1.94 | 26.51 ± 3.28 | 3.41 ± 0.48 | 24.88 ± 1.69 |
| –4 | 21 | 42.62 ± 1.47 | 26.17 ± 1.65 | 3.57 ± 0.35 | 24.55 ± 0.69 |
| –3 | 30 | 42.44 ± 1.68 | 25.87 ± 1.60 | 3.42 ± 0.26 | 24.20 ± 0.66 |
| –2 | 71 | 42.55 ± 1.51 | 25.94 ± 2.19 | 3.41 ± 0.32 | 23.74 ± 0.81 |
| –1 | 245 | 42.27 ± 1.40 | 25.97 ± 1.84 | 3.26 ± 0.30 | 23.40 ± 0.69 |
| 0 | 818† | 42.01 ± 1.41 | 25.98 ± 1.86 | 3.19 ± 0.30 | 23.14 ± 0.74 |
| 1 | 644 | 41.89 ± 1.45 | 25.96 ± 1.80 | 3.09 ± 0.29 | 22.89 ± 0.72 |
| 2 | 105 | 41.71 ± 1.61 | 25.81 ± 1.86 | 2.95 ± 0.27 | 22.64 ± 0.78 |
| 3 | 19 | 41.96 ± 1.74 | 26.33 ± 2.06 | 3.01 ± 0.27 | 22.11 ± 0.87 |
| 4 | 12 | 41.94 ± 1.88 | 26.56 ± 1.86 | 2.92 ± 0.25 | 21.82 ± 0.80 |
| Corr with SE\* | | –0.111 (< 0.001) | –0.085 (< 0.001) | –0.313 (< 0.001) | –0.570 (< 0.001) |
| Corr with AL\* | | 0.569 (< 0.001) | –0.537 (< 0.001) | 0.491 (< 0.001) | – |
| Parameters defined in Table 1. \*Pearson correlation, p < 0.05/4 (Bonferroni correction) indicates significance. †Lower value than in the paper as SE = + 0.5D was consider as part of the +1D bin here. | | | | | |

## Slide 14
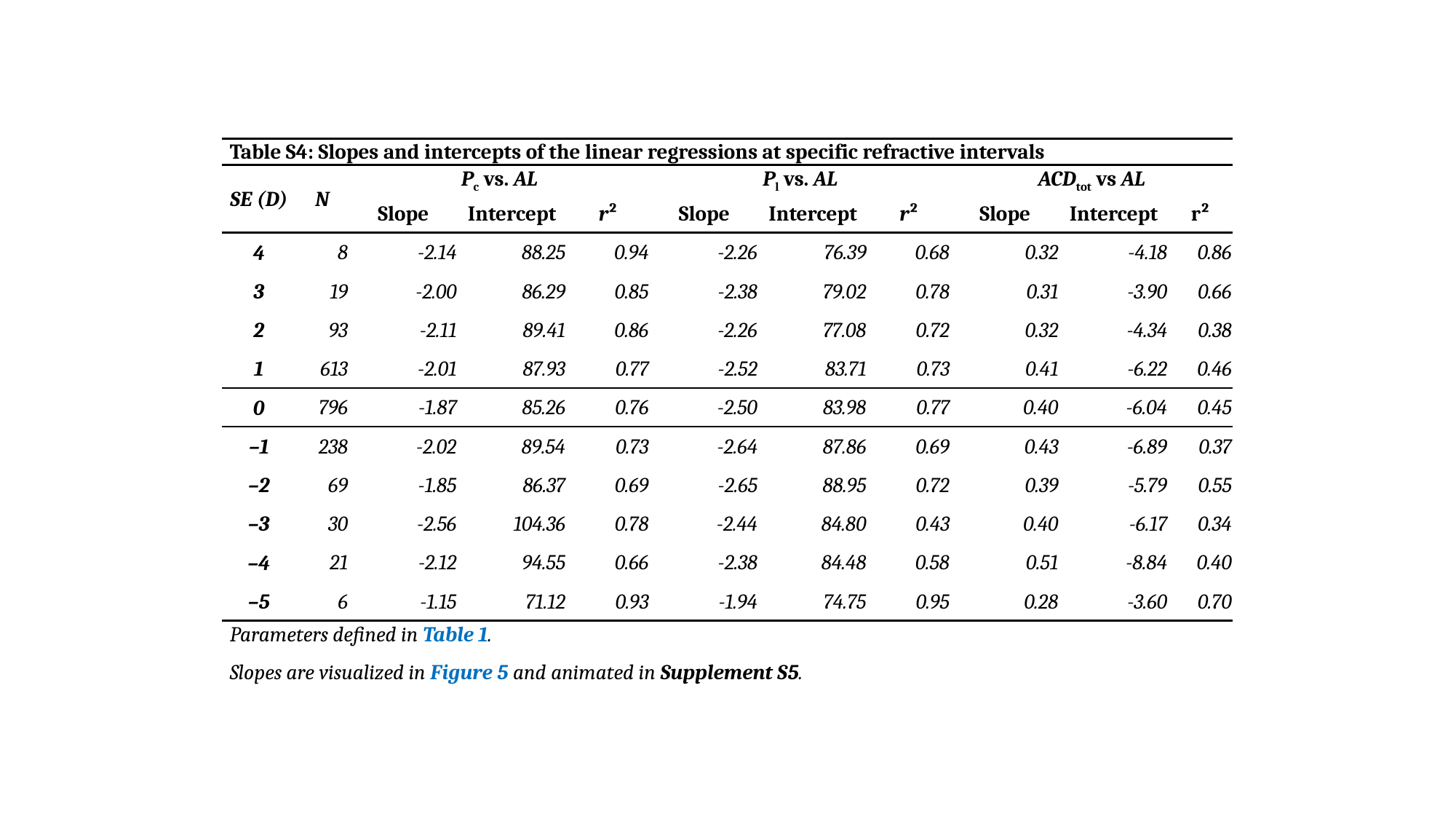

| Table S4: Slopes and intercepts of the linear regressions at specific refractive intervals | | | | | | | | | | |
| --- | --- | --- | --- | --- | --- | --- | --- | --- | --- | --- |
| SE (D) | N | Pc vs. AL | | | Pl vs. AL | | | ACDtot vs AL | | |
| | | Slope | Intercept | r² | Slope | Intercept | r² | Slope | Intercept | r² |
| 4 | 8 | -2.14 | 88.25 | 0.94 | -2.26 | 76.39 | 0.68 | 0.32 | -4.18 | 0.86 |
| 3 | 19 | -2.00 | 86.29 | 0.85 | -2.38 | 79.02 | 0.78 | 0.31 | -3.90 | 0.66 |
| 2 | 93 | -2.11 | 89.41 | 0.86 | -2.26 | 77.08 | 0.72 | 0.32 | -4.34 | 0.38 |
| 1 | 613 | -2.01 | 87.93 | 0.77 | -2.52 | 83.71 | 0.73 | 0.41 | -6.22 | 0.46 |
| 0 | 796 | -1.87 | 85.26 | 0.76 | -2.50 | 83.98 | 0.77 | 0.40 | -6.04 | 0.45 |
| –1 | 238 | -2.02 | 89.54 | 0.73 | -2.64 | 87.86 | 0.69 | 0.43 | -6.89 | 0.37 |
| –2 | 69 | -1.85 | 86.37 | 0.69 | -2.65 | 88.95 | 0.72 | 0.39 | -5.79 | 0.55 |
| –3 | 30 | -2.56 | 104.36 | 0.78 | -2.44 | 84.80 | 0.43 | 0.40 | -6.17 | 0.34 |
| –4 | 21 | -2.12 | 94.55 | 0.66 | -2.38 | 84.48 | 0.58 | 0.51 | -8.84 | 0.40 |
| –5 | 6 | -1.15 | 71.12 | 0.93 | -1.94 | 74.75 | 0.95 | 0.28 | -3.60 | 0.70 |
| Parameters defined in Table 1. Slopes are visualized in Figure 5 and animated in Supplement S5. | | | | | | | | | | |

## Slide 15
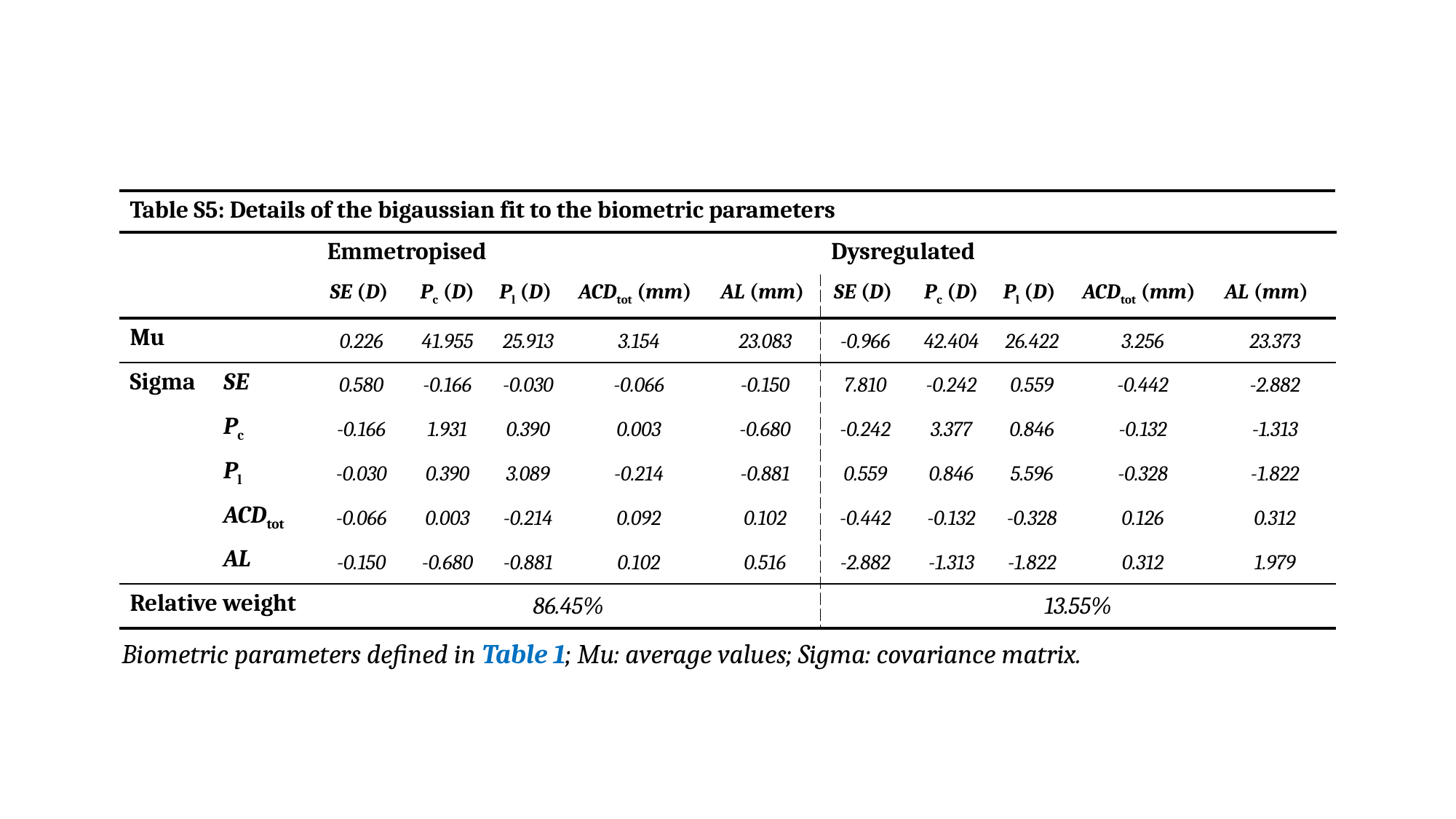

| Table S5: Details of the bigaussian fit to the biometric parameters | | | | | | | | | | | |
| --- | --- | --- | --- | --- | --- | --- | --- | --- | --- | --- | --- |
| | | Emmetropised | | | | | Dysregulated | | | | |
| | | SE (D) | Pc (D) | Pl (D) | ACDtot (mm) | AL (mm) | SE (D) | Pc (D) | Pl (D) | ACDtot (mm) | AL (mm) |
| Mu | | 0.226 | 41.955 | 25.913 | 3.154 | 23.083 | -0.966 | 42.404 | 26.422 | 3.256 | 23.373 |
| Sigma | SE | 0.580 | -0.166 | -0.030 | -0.066 | -0.150 | 7.810 | -0.242 | 0.559 | -0.442 | -2.882 |
| | Pc | -0.166 | 1.931 | 0.390 | 0.003 | -0.680 | -0.242 | 3.377 | 0.846 | -0.132 | -1.313 |
| | Pl | -0.030 | 0.390 | 3.089 | -0.214 | -0.881 | 0.559 | 0.846 | 5.596 | -0.328 | -1.822 |
| | ACDtot | -0.066 | 0.003 | -0.214 | 0.092 | 0.102 | -0.442 | -0.132 | -0.328 | 0.126 | 0.312 |
| | AL | -0.150 | -0.680 | -0.881 | 0.102 | 0.516 | -2.882 | -1.313 | -1.822 | 0.312 | 1.979 |
| Relative weight | | 86.45% | | | | | 13.55% | | | | |
Biometric parameters defined in Table 1; Mu: average values; Sigma: covariance matrix.
